# The Frequency and Context of Snacking among Children: An Objective Analysis Using Wearable Cameras

**DOI:** 10.3390/nu13010103

**Published:** 2020-12-30

**Authors:** Ryan Gage, Martin Girling-Butcher, Ester Joe, Moira Smith, Cliona Ni Mhurchu, Christina McKerchar, Viliami Puloka, Rachael McLean, Louise Signal

**Affiliations:** 1 Department of Public Health, University of Otago, Wellington 6242, New Zealand; girma304@student.otago.ac.nz (M.G.-B.); joees647@student.otago.ac.nz (E.J.); moira.smith@otago.ac.nz (M.S.); viliami.puloka@otago.ac.nz (V.P.); louise.signal@otago.ac.nz (L.S.); 2 National Institute for Health Innovation, University of Auckland, Auckland 1142, New Zealand; c.nimhurchu@auckland.ac.nz; 3 Department of Population Health, University of Otago, Christchurch 8140, New Zealand; christina.mckerchar@otago.ac.nz; 4 Department of Preventive and Social Medicine, University of Otago, Dunedin 9016, New Zealand; rachael.mclean@otago.ac.nz

**Keywords:** snacking, eating, children, obesity, wearable cameras

## Abstract

Snacking is a common eating behaviour, but there is little objective data about children’s snacking. We aimed to determine the frequency and context of children’s snacking (*n* = 158; mean age = 12.6 years) by ethnicity, gender, socioeconomic deprivation and body mass index (BMI) children. Participants wore wearable cameras that passively captured images of their surroundings every seven seconds. Images (*n* = 739,162) were coded for snacking episodes, defined as eating occasions in between main meals. Contextual factors analysed included: snacking location, food source, timing, social contact and screen use. Rates of total, discretionary (not recommended for consumption) and healthful (recommended for consumption) snacking were calculated using negative binomial regression. On average, children consumed 8.2 (95%CI 7.4, 9.1) snacks per day, of which 5.2 (95%CI 4.6, 5.9) were discretionary foods/beverages. Children consumed more discretionary snacks than healthful snacks in each setting and at all times, including 15.0× more discretionary snacks in public spaces and 2.4× more discretionary snacks in schools. Most snacks (68.9%) were sourced from home. Girls consumed more total, discretionary and healthful snacks than boys, and Māori and Pacific consumed fewer healthful snacks than New Zealand (NZ) Europeans. Results show that children snack frequently, and that most snacking involves discretionary food items. Our findings suggest targeting home buying behaviour and environmental changes to support healthy snacking choices.

## 1. Introduction

Snacking is a common eating behaviour. However, measuring its contribution to the diet presents several challenges for researchers. Most importantly, there is no fixed definition of snacking. As highlighted in recent reviews [1,2], the most common definition classifies snacking episodes as food/beverages consumed in between main meals. However, even among studies following this definition, approaches to quantifying these foods and beverages vary. Examples include time-based approaches, where snacks are differentiated from meals based on time (with meals generally including the largest eating episode falling within pre-specified time windows); participant-identified approaches, where participants themselves distinguish snacks from meals; and food-based classification, which differentiates snacks from meals based on nutritional profile, e.g., energy density [3].

Irrespective of the approach used, however, previous evidence confirms that snacking is a highly frequent eating behaviour in many countries. Based on participant-identified cross-country surveys, Wang et al. (2018) found that most children aged 9–13 years in Australia, China, Mexico and the United States (US) consume at least one snack a day [4]. The authors found that snacking rates were highest in Australia, where most children consumed at least four snacks a day, and snacking provided one-third of total energy intake [4]. Another study utilizing a time-based measure found that Tongan children aged 11 years consume an average of 5.8 snacks per day [5].

High rates of snacking among children highlight the need for clear nutritional guidance around snacking behaviour, particularly given the global childhood obesity epidemic. However, developing recommendations around snacking is not straightforward, as it may present both benefits and harms to health. On the one hand, snacking on healthful foods and beverages (e.g., fruit and nuts) contributes important micronutrients to the diet, and has been associated with weight loss or weight maintenance [2]. Indeed, while nutritional guidance on snacking varies globally, several jurisdictions recommend that children consume healthful snacks to meet daily energy and nutrient requirements [6]. On the other hand, evidence suggests that most snacks consumed by children are highly-processed and high in salt, fat or sugar, such as confectionery and crisps [7]. There is evidence that snacking on such foods is associated with increased energy intake, poor nutrient intake and weight gain [2,8].

To promote healthier snacking behaviours, research on the determinants of snacking is needed. Recent reviews have identified numerous factors that influence snacking behaviour [1,2]. Individual-level factors include gender [9], age [10], distractions (e.g., TV viewing) [11] and stress [12,13]. Environmental and social factors also play a role, including the availability of unhealthy foods in homes, schools and neighbourhoods [14,15,16], food prices [17], food and beverage marketing [18], convenience store access [12,19], parental modelling [14] and peer social norms [20].

However, the literature on snacking has several deficiencies. Most studies have used participant self-report methods, mainly 24 h recalls and food diaries [2]. These methods are vulnerable to recall bias and have been shown to underestimate eating episodes, particularly snacking [21]. Additionally, little is known about the context that underpins snacking, such as eating locations, food sources and time of consumption [14,22]. Addressing these limitations could help in more accurately identifying the contribution of snacking to children’s dietary intake and help identify strategies to support healthy snacking behaviour (e.g., by identifying key settings for intervention).

There has been limited research on children’s snacking in New Zealand (NZ). The last national survey of child nutrition in NZ, conducted in 2002, did not differentiate snacks from meals [23]. Other research related to child snacking in NZ has been limited to secondary analyses of the 2002 survey data [24,25] and a small study (*n* = 44) of five-year-old children [26]. Filling this gap in knowledge is important given high rates of childhood obesity in NZ (second highest among Organisation for Economic Co-operation and Development (OECD) Member countries), alongside the disproportionate burden of obesity and food insecurity experienced by Māori and Pacific children [27,28].

Wearable cameras offer a valuable opportunity to study the frequency and context of snacking behaviours. Wearable cameras passively capture images of the wearer’s surroundings at fixed intervals, enabling an objective analysis of their lived experience. Two studies found that wearable cameras enhance the accuracy of self-reported dietary intake [29,30]. Wearable cameras have recently been used as a sole methodology to investigate Tongan children’s eating episodes [5], NZ children’s beverage consumption [31] and eating and drinking behaviours during transport journeys among young Australian adults [32].

The purpose of this study was to explore the frequency and context of snacking among children who participated in the 2014/15 NZ Kids’Cam wearable camera project [33]. Our specific aims were to:

1. Identify rates of total, discretionary and healthful snacking among children per day.
2. Determine the context of snacking episodes relating to food group, eating location, source, time of consumption, social contact and screen use.
3. Examine the association between sociodemographic characteristics (gender, ethnicity and household socioeconomic deprivation) and body mass index (BMI) and total, discretionary and healthful snacking rates.

## 2. Materials and Methods

### 2.1. The Kids’Cam Project

Kids’Cam was a cross-sectional observational study of 168 children aged 11–13 years in the Wellington region of NZ [33]. The study aimed to explore the world in which children live, using a combination of wearable camera and GPS technology. Children were recruited at random from 16 randomly-selected schools in the Wellington region, using a two-stage, stratified sampling procedure detailed elsewhere [33]. In the first stage, schools were randomly sampled based on probability-proportional-to-size stratified random sampling by school decile (low decile = 1–3, medium decile = 4–7, high decile = 8–10) and student ethnicity (Māori, Pacific and NZ European (NZE)). In the second stage, a maximum 15 eligible children, stratified by ethnicity, were randomly selected from each consenting school, and invited orally to participate in the study. The first six who returned signed consent forms (including parental consent) were selected to take part from this number. The sampling strategy generated nine sampling strata.

In a briefing session, selected participants were asked to wear a camera and GPS device on lanyards around their neck for four consecutive days (Thursday to Saturday) for all waking hours. Children were instructed to remove the camera in situations where privacy could be expected, if they felt uncomfortable, when swimming or playing vigorous sport or if requested by others [33]. The wearable cameras captured images of their surroundings every seven seconds. The study was conducted in accordance with the Declaration of Helsinki; ethical approval was obtained from the University of Otago Human Ethics Committee (Health) (13/220) to study all aspects of children’s lives relevant to public health. All participating children, parents and schools signed written consent to participate in the study. Further methodological details are published elsewhere [33,34].

In this ancillary study of snacking behaviour, we included participants who captured useable image data on a Thursday and/or a Saturday (i.e., those with eligible data on at least one of these days) to capture both school time and leisure time eating behaviours. ‘Uncodable’ images were excluded, including blurry and blocked images and images captured when cameras were turned on but not worn.

### 2.2. Methods for Coding Snacking Behaviour

A coding protocol was developed to guide the coding of children’s snacking behaviour (Supplementary Material S1). Coding definitions were adapted from a previous wearable camera project on Tongan children’s eating behaviour [5], which we piloted and refined for use in NZ. These definitions (described below, with further detail in Supplementary Material S1) were subjected to an inter-rater coding reliability test of 11,662 images, on which three coders achieved >90% agreement for all codes.

Snacking was defined as any food or beverage, excluding water, consumed in-between main meals (breakfast, lunch or dinner). We excluded water because piloting revealed that frequent sipping of water in drink bottles, particularly during class-time at school (when consumption of other foods/beverages is restricted), would greatly overinflate snacking rates. We distinguished snacks from meals using an established time-based approach [3], in which the largest eating episodes occurring between 06:00–10:00, 12:00–15:00 and 17:00–20:00 h were considered meals. Therefore, snacking episodes included smaller eating episodes occurring within these times and eating episodes falling outside these times. Main meals were considered finished when five minutes (approximately 43 images) passed after participants were observed eating food/beverages in that meal. We tested a variation to this time-based approach using sensitivity analyses (described below).

**Figure 1:**
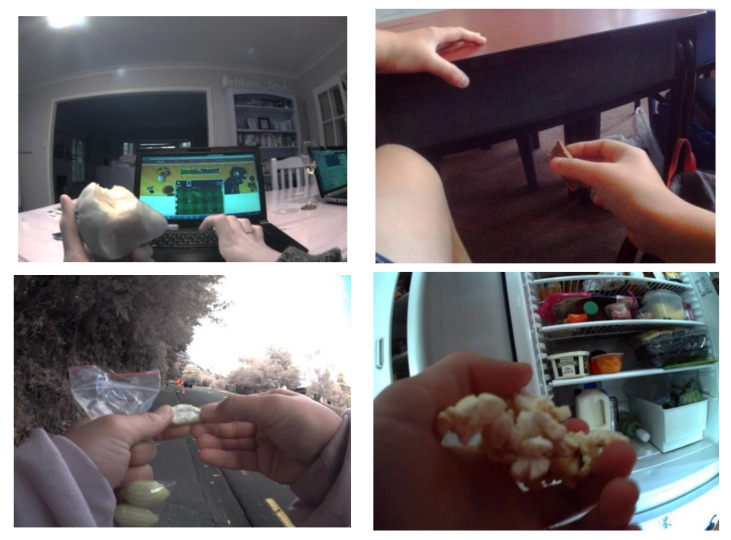
(**Top left**) snacking on pear at home; (**Top right**) snacking on chocolate in school; (**Bottom** **left**) snacking on candy on street; (**Bottom right**) snacking on popcorn at home.

The coding process involved viewing each sequential image, with frequency and context codes entered in a pre-formatted Excel spreadsheet. Coding was completed by two researchers in 2019/20. Snacking episodes were coded when one of two conditions were met: (1) The image sequence showed participants lifting a snack food or beverage towards their face; or (2) The image sequence showed participants handling a snack food or beverage, accompanied by evidence that the product was decreasing in quantity. Figure 1 shows several examples of snacking episodes. Like previous wearable camera studies [5,31], we considered snacking episodes as ‘complete’ when five minutes (approximately 43) images passed without further consumption evidence. After this time, evidence of further snacking was considered to be new snacking episodes.

Snacks were coded by food/beverage category and further classified as discretionary, healthful or ‘other’, based on recommendations in NZ’s Food and Nutrition Guidelines [35]. Discretionary snacks were defined as foods/beverages that are not recommended for children’s consumption due to contents high in salt, fat or sugar. Discretionary food groups included confectionery, cookies/cakes, diet drinks, fast food, ice cream, cereals with >15 g/100 g total sugars (determined using codes developed previously: [34]), milk products (dairy food and yoghurt) with >10 g/100 g total sugar (determined using codes developed previously: [34]) savoury bakery items, processed meat, snack foods and sugary drinks/juices. Healthful snacks were defined as foods/beverages that are recommended for consumption by children. Healthful food groups included breads/cereals (including cereals <15 g/100 g total sugars [34]) fruit, meat/meat alternatives, healthy milk products (<10 g/100 g total sugars [34]) and vegetables. Snacks that did not meet the definition of discretionary or healthful were classified as ‘other’ (e.g., tea and coffee with no added sugar). Snacks that could not be identified in the images were classified as undetermined.

Mixed dishes were defined as dishes with mixed ingredients and/or multiple items. Mixed dishes containing only discretionary or healthful ingredients were classified as discretionary or healthful, respectively. Mixed dishes containing combinations of discretionary and healthful foods were classified as ‘other’ (e.g., ice cream and fruit). Mixed dishes containing unknown or indistinguishable ingredients were classified as ‘undetermined’.

### 2.3. Contextual Factors

Contextual factors evaluated included, eating location, source, timing, screen use and social interaction.

Eating locations included homes, schools and public spaces (subcategories of public spaces included: recreation/sport spaces, food outlets, other retail stores and ‘other’). Eating locations were determined based on the surroundings shown in the image and contextual cues, based on definitions developed in previous Kids’Cam research [34]. If a snacking episode occurred in multiple locations, the only setting coded was that in which children spent the most time.

The source of a snack refers to the setting (e.g., food outlet) or person (e.g., adult, child) from which a child obtained the snack. Snacks sourced from food outlets or school canteens were coded as ‘purchased’ if coders observed evidence that the participant or another person purchased it through visual evidence of a transaction (e.g., exchange of money).

Timing refers to the time of day in which a snack was consumed. Time data were obtained from image-time stamps, which were recorded to the nearest second. We categorised timing by the hour of day and by morning (06:00–11:59 h), afternoon (12:00–17:59 h) and evening (18:00–23:59 h).

During snacking, social contact was determined if there was visible evidence that surrounding people were associated with the participant. We assumed that other children were always present in school settings (a decision based on method piloting). During a snacking episode, social contact was only coded if there was evidence of social contact in more than 50% of images in that episode.

Screen use included interactions with screens (e.g., playing games on tablets) and television watching (e.g., when televisions were on in the background). Similar to social contact, screen use during a snacking episode was only coded if screens appeared to be used in more than 50% of the images in that episode. This generally excluded brief interactions with mobile devices, e.g., sending a text message.

### 2.4. Statistical Analysis

Statistical analyses were conducted in Stata/16. Counts of total, discretionary and healthful snacking episodes were collated by child for the two-day observation period. Rates of snacking were calculated using negative binomial regression, as appropriate for over-dispersed count-based numerator data [36]. The numerator for these rates was snacking episodes, and the denominator was total image recording time (calculated by multiplying image counts by the median image capture rate of seven seconds). Similar to previous wearable camera analyses [5,31,34,37,38,39], we rescaled these rates to represent the frequency of snacking per 10 h of recording time (an approximate ‘day’). Given the stratified sampling of schools and children (described above) [34], we applied inverse sampling weights using Stata’s svy weights and associated weighting options. Rates of snacking are presented as means of snacking per 10 h day with 95% confidence intervals.

To describe the context of snacking behaviour, rates of total, discretionary and healthful snacking (with 95% CIs) are reported for subcategories of: food group, eating locations, food source, time of day, screen use and social contact. While reporting fractional values for snacks is not intuitive at the individual level (e.g., it would be impossible under our coding method to consume 0.5 of a snack), snacking is reported to one decimal place to provide more meaningful comparisons across groups.

We used multivariate negative binomial regression models to evaluate differences in total, discretionary and healthful snacking by sex, ethnicity, household socioeconomic deprivation (NZiDep) categories ‘lower deprivation’ (groups 1–3) and ‘higher deprivation’ (groups 4–5) [40] and BMI categories ‘not overweight’ and ‘overweight’. Adjusted rate ratios (aRRs) were mutually adjusted for gender, ethnicity, BMI and NZiDep.

Subgroup differences in snacking by eating location were examined by substituting overall snacking and observation time counts with counts specific to each location (homes, schools and public spaces). We made this adjustment because Kids’Cam data showed several subgroup differences in image capture by setting (e.g., boys spent more time in public spaces than girls).

Given the importance of timing for distinguishing snacks from meals, we conducted a sensitivity analysis in which all eating episodes consumed between 06:00–10:00, 12:00–15:00 and 17:00–20:00 h were considered meals, rather than the largest eating episode only. This approach has been used previously [41] and is more conservative than the time-based approach used in our baseline calculation [3].

## 3. Results

### 3.1. Sample Characteristics

The study sample included 158 participants (Table 1). We excluded ten original study participants (6.0%) who captured no data on Thursday or Saturday. Just over half (52.5%) of participants were female. There were proportionately fewer overweight children (43.7%) than not overweight children (57.3%), and children living in conditions of higher socioeconomic deprivation (32.0%) than children living in conditions of lower socioeconomic deprivation (68.0%). The ethnic distribution was 41.1% NZ European, 36.7% Māori and 22.2% Pacific.

Participants captured 4678 images each, on average, over the two-day observation period, for a total of 739,162 images. Most images (96.1%) were codable; the remaining 3.9% were excluded owing to blurriness, blockage, or capture when cameras were turned on but not worn. Mean observation time was 8.7 h (95% CI 9.0, 9.4)/participant. Observation time was higher for Thursdays (6.1 h, 95% CI 5.7, 6.5) than Saturdays (2.7 h, 95% CI 2.4, 3.1).

### 3.2. Snacking Frequency and Snacking Food Choices

Most children (94.9%) consumed at least one snack in the observation period. Table 2 presents rates of total, discretionary and healthful snacking per 10 h by specific context categories. On average, participants consumed 8.2 snacks (95% CI: 7.4, 9.1) per 10 h. Participants consumed more than twice as many discretionary snacks (5.2 per 10 h) as healthful snacks (2.1 per 10 h). Common discretionary snacks included confectionary (1.3 episodes per 10 h), snack foods (1.2 episodes per 10 h), cookies/cakes (1.0 episodes per 10 h) and sugary drinks/juices (1.0 episodes per 10 h). The most commonly consumed healthful snacks were fruit (0.7 episodes per 10 h) and breads/cereals (0.7 episodes per 10 h), which accounted for 71.9% of healthy snack foods. Children rarely snacked on nuts (<0.1 episodes per 10 h) and vegetables (<0.1 episodes per 10 h). Fewer than one in 10 (8.5%) snacks were of an undetermined food group.

Mixed dishes accounted for 7.9% of snacking episodes. Most mixed dishes (44%) had contents that could not be determined; 13% contained only healthful items (e.g., cereal and milk); 11% contained only discretionary items (e.g., ice cream and jelly); 28% contained both discretionary and healthful items (e.g., sausages in bread).

### 3.3. Eating Location

**Figure 2:**
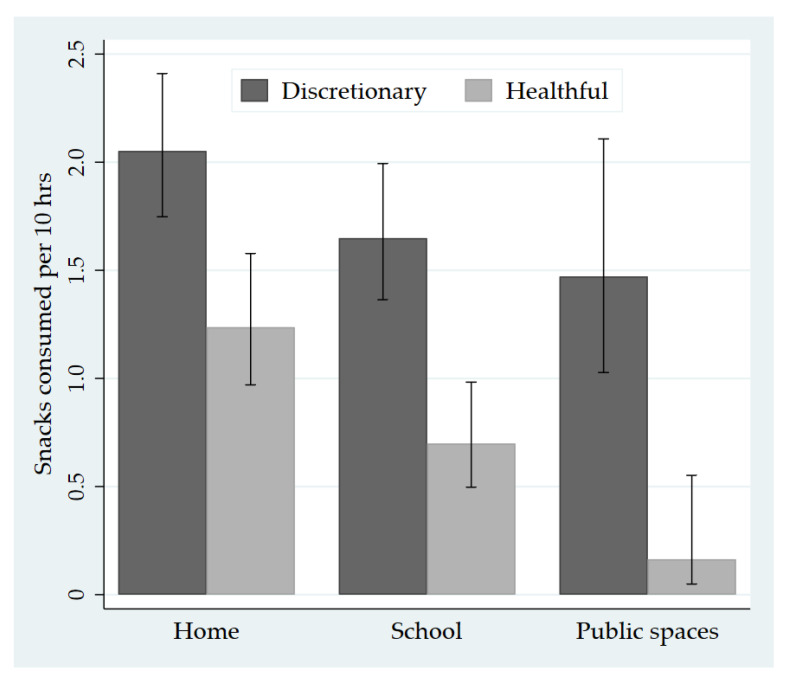
Children’s consumption of discretionary and healthful snacks per 10 h by eating location.

Children ate most snacks at home (47.5%; 3.9 per 10 h), followed by at school (31.7%; 2.6 per 10 h) and in public spaces (21.9%; 1.8 per 10 h). Compared with healthful snacking rates, children’s consumption of discretionary snacks was 15.0 times higher in public spaces, 2.4 times higher at school and 1.7 times higher at home (Figure 2). Children ate discretionary snacks at a higher rate than healthful snacks in each type of public space, ranging from 7.0 times higher in food outlets to 16.8 times higher in sport/recreation spaces.

### 3.4. Sources of Snacks

Children sourced most snacks from home (68.9%). Children sourced proportionately fewer discretionary snacks from home (62.7%) than healthful snacks (81.7%). Children only sourced 4.8% of total snacks from school, despite eating 31.7% of total snacks at school (schools in NZ do not typically provide meals, but some do have food for sale). More than half the snacks that children sourced from school (51.9%) were discretionary foods. Food outlets were a common source of discretionary snacks (16.8% of total), but not healthful snacks (3.0% of total). Most snacks (60.0%) sourced from food outlets were from convenience stores or fast-food outlets. Twenty-six children (17.7%) purchased and consumed a snack, all of which were discretionary. Thirty-one children consumed a snack purchased by an adult (19.6%), of which 81.6% were discretionary.

### 3.5. Timing

**Figure 3:**
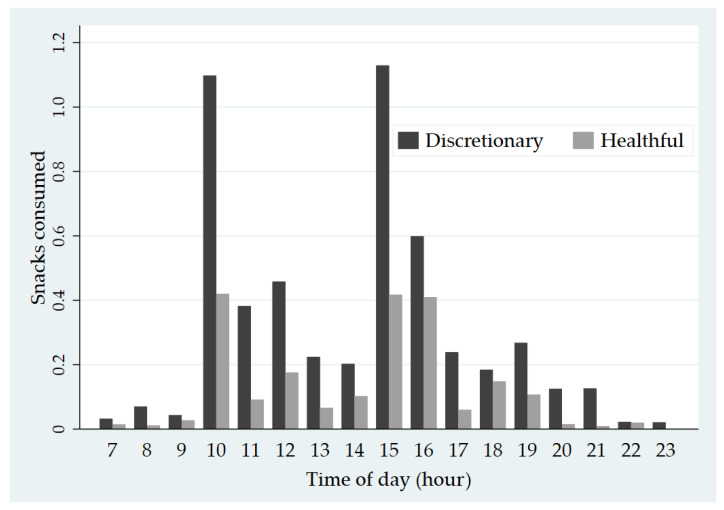
Rates of discretionary and healthful snacking by the hour.

Most snacks (55.9%; 4.6 per 10 h) were consumed in the afternoon period between 12:00 and 17:59 h. Proportionately fewer snacks were consumed in the morning between 06:00 and 11:59 h (29.7%; 2.4 per 10 h) and evening between 18:00 and 23:59 h (14.0%; 1.2 per 10 h). Figure 3 summarizes rates of discretionary and healthful snack consumption by hour. Children ate more discretionary snacks than healthful snacks across all hours. The highest rates of snacking were observed in the hours of 10am, 3pm and 4pm.

### 3.6. Screen Use and Social Contact

Children used screens during one in four (24.0%) snacking episodes. There was no evidence that screen use was associated with the nutritional quality of snacks; children used screens over 50% of the time during 24.1% of discretionary snacking episodes and 25.1% of healthful snacking episodes. Children had social contact during 75.5% of snacking episodes (77.3% for discretionary snacks and 72.8% for healthful snacks). Adults were present in nearly one-third of snacking episodes (31.5%). Adults were present in more public space snacking episodes (54.2%) than home snacking episodes (37.7%) and school snacking episodes (6.3%). The lower presence of adults in schools was explained by the consumption of snacks in outdoor areas during morning tea (recess) and lunch breaks, where teachers were rarely near children.

### 3.7. Differences in Snacking Episodes by Sociodemographic Characteristics and BMI

Table 3 shows the mean rates and rate ratios (95% CI) of total, discretionary and healthful snacking by sociodemographic characteristics and BMI. In the adjusted calculations, boys consumed significantly fewer total snacks (aRR = 0.79), discretionary snacks (aRR = 0.80) and healthful snacks (aRR = 0.71) than girls. Ethnicity was not associated with total snacks and discretionary snack consumption, but Māori and Pacific children consumed significantly fewer healthful snacks than NZ European children (aRR for Māori = 0.54; aRR for Pacific = 0.58). There was no association between children’s BMI and their consumption of total, discretionary and healthful snacks. There was some evidence that overweight/obese children consumed fewer healthful snacks than non-overweight children, but this difference was not significant in the adjusted model (Table 3).

When snacking episodes were delineated by eating location, we found strong evidence of gender differences in snacking (Supplementary Material S2). Compared with girls, boys ate less than half as many total (aRR = 0.45) and discretionary snacks (aRR = 0.42) in public spaces, and less than half as many healthful snacks in schools (aRR = 0.48). There was evidence that Māori and Pacific children consumed less healthful snacks at home and school than NZ European children, which is consistent with ethnic differences in healthful snacking across all settings combined (Supplementary Material S2).

### 3.8. Sensitivity Analysis

When applying the conservative scenario in which all eating episodes consumed between 06:00–09:00, 12:00–15:00 and 17:00–20:00 h are considered meals, the total snacking rate decreased 32.9% from 8.2 per 10 h to 5.5 per 10 h. This scenario excluded, on average, 0.2 eating episodes between 06:00–09:00, 1.3 eating episodes between 12:00–15:00 and 1.2 eating episodes between 17:00–20:00, for a total of 2.7 eating episodes. Discretionary and healthful snacks accounted for a similar proportion of total snacks under each scenario (Baseline scenario: 63.6% discretionary, 25.5% healthful; Conservative scenario: 64.5% discretionary; 25.6% healthful).

## 4. Discussion

Children in this study consumed, on average, 8.2 snacks per 10-h day. Although snacking provides healthful food consumption opportunities, most snacks consumed by children were discretionary (63.6%; 5.2 per 10 h). We found higher rates of discretionary snacking than healthful snacking in all settings and hours of day, highlighting the need to promote healthy snacking behaviours among NZ children.

The high rate of snacking observed, particularly discretionary snacking, is consistent with previous research on children of this age. In a similar wearable camera study, Veatupu et al. (2019) found that Tongan children aged 11 years consume four unhealthy snacks a day and 1.8 healthful snacks a day [5]. Using participant-identified surveys, Wang et al. (2018) found that more than half of Australian children aged 9–13 years consumed at least four snacks a day, with most children in the US and Mexico consuming 2–3 snacks a day [4]. Regarding snack food choices, the patterns observed in our study resemble those reported in Australia, the US and Tonga, where confectionery, cookies/cakes, salty snacks and fruit are frequently consumed snacks [4,5].

Research on snacking must carefully consider how snacks are distinguished from meals. In this study, we used an established time-based approach [3], in which the largest eating episode occurring between 06:00–09:00, 12:00–15:00 and 06:00–09:00 h were considered meals. However, we also tested a stricter assumption in which all eating episodes occurring between these times were classified as meals. The conservative approach resulted in a 32.9% reduction in snacking frequency (5.5 per 10 h compared with 8.2 snacks per 10 h) but did not influence the proportion of discretionary and healthful snacks included. While the strict scenario did not alter our conclusions (i.e., snacking was highly prevalent in both scenarios), the sensitivity of the snacking rate to timing has important implications for future studies using a time-based approach. It stresses the importance of explicitly stating the time windows used to distinguish snacks from meals. Where possible, sensitivity analyses of key assumptions should be employed (e.g., by modifying time windows as presented here), or by comparison with other approaches (e.g., participant identified).

We found that the nutritional quality of snacks depended on eating location. Children consumed 15.0 times more discretionary snacks than healthful snacks in public spaces, compared with 2.4 times more in schools and 1.7 times more in homes. This was despite a higher presence of adults during public space snacking episodes, suggesting that adult supervision was not protective. Furthermore, all the snacks that children purchased from public spaces were discretionary, alongside 51.9% of snacks sourced from school. These findings build on previous concerns about obesogenic environments in NZ, including unhealthy marketing in public spaces [34], high availability and access to unhealthy foods [42,43] and the lack of mandatory school food policies in NZ primary and secondary schools [43].

However, while unhealthy snacking behaviours were apparent in schools and public spaces, children sourced most snacks from home (68.9%). This is consistent with previous research [5,24]. These findings highlight the importance of targeting home-buying behaviour to improve children’s snacking behaviour. Relevant interventions include the provision of appropriate and context-specific nutrition information, restrictions on food and beverage marketing, development of effective, standardised nutrient labelling systems and taxes on sugar-sweetened beverages [44,45].

We found that girls ate more total, discretionary and healthful snacks than boys, which was largely driven by differences in discretionary snacking in public spaces. There has been limited research on gender differences in snacking frequency, and research on setting-specific differences is particularly sparse. Our findings contrast with a study of Dutch adolescents aged 12–17 years, which found higher snacking rates among boys [9]. It also contrasts with the findings of the 2002 National Children’s Nutrition Survey, which, although not focusing on snacking specifically, found little differences in the foods and beverages consumed by gender (e.g., in the food category, ‘sweets and snacks’) [23].

Several reasons may explain the higher snacking rate among girls. First, it is possible that girls have a greater propensity to consume unhealthy foods and beverages in public spaces. This has been suggested previously by Xin et al. (2019), who found that girls, if given access to convenience stores, are more likely to engage in unhealthy eating behaviours [19]. Second, although we found higher snacking frequency among girls, it is possible that boys consumed snacks in larger portion sizes. The United Kingdom National Diet and Nutrition Survey, for example, found that boys consumed larger portions of energy-dense snacks than girls [46]. More research could be warranted to explore reasons for gender differences in eating patterns, particularly in settings outside home.

### Study Strengths and Limitations

A key strength of our study was the objective method of data collection using wearable cameras. Most studies of snacking behaviour have used self-report methods, which are prone to self-report bias and have been shown to underestimate snacking episodes [21]. Wearable cameras also enabled us to explore the contextual factors that underpin snacking behaviour. The findings address a knowledge gap in NZ, given that the last national survey on children’s nutrition was conducted in 2002 and did not differentiate between snacks and meals [23].

Some limitations should be noted, however. First, we could not determine the quantity of snack food consumed, nor the contribution of snacking to children’s energy and nutrient intake. Future studies using wearable cameras could address this limitation by pairing the devices with participant self-report methods such as food diaries and interviews. Second, although wearable cameras can identify eating occasions missed by self-report [21], it is likely that some snacking episodes were missed due to camera removal or uncodable images. While most images (96.1%) were codable and the passive method of data collection had a relatively small participant burden [33], average recording time was less than two full waking days, suggesting that camera removal took place. Higher image capture on Thursday than Saturday may also bias our findings towards in-school snacking behaviour relative to leisure-time snacking behaviour. However, since children spend five days a week at school, this makes up for fewer recording hours on Saturday.

Finally, to ensure that our coding process was reproducible, we limited the analysis to factors that could be reliably coded (i.e., with >90% agreement on inter-rater reliability tests). This allowed a basic determination of screen use and social contact, and the relationship between screens and social contact and the nutritional quality of snacks (for which we found no association). However, the approach was unsuitable for capturing some deeper determinants of snacking, such as parent behaviours (e.g., role modelling) and peer social norms [20]. Further, our time-based measure of snacking did not capture participants’ views about snacking behaviour. For example, it is possible that participants may have regarded some eating episodes as meals, not snacks, and vice versa. Given this, combining wearable cameras methods with participant-identified methods, such as food diaries or photo-elicitation, may enable a more detailed analysis of snacking behaviour. However, while wearable cameras have a relatively small participant burden, researchers must be aware that the coding of images for eating episodes takes substantial time (approximately one hour per hour of photographs).

## 5. Conclusions

In this study, wearable cameras enabled objective analysis of children’s snacking behaviours, including frequency and the contextual factors that underpin it, in a country with limited data on snacking behaviours. Children in this study consumed an average of 8.2 snacks per day, most of which were discretionary foods. Our findings highlight the importance of actions in this arena to improve children’s dietary behaviours. Given that children sourced most snacks from home, our study supports holistic interventions targeting home purchasing behaviour. High rates of discretionary snacking in public spaces and schools also suggest the need for environmental changes to support healthy choices.

## Figures and Tables

**Table 1:** Sample characteristics.

Sociodemographic Variable and Group	*n*	%
Total	158	100
**Gender**		
Female	83	52.5
Male	75	47.5
Ethnicity		
NZ European	65	41.1
Māori	58	36.7
Pacific	35	22.2
**Household socioeconomic deprivation**		
Lower (NZiDep 1–3)	104	68
Higher (NZiDep 4–5)	49	32
**Age (years)**		
11	13	8.2
12	115	72.8
13	24	15.2
14	1	0.6
Mean (SD)	12.6 (0.5)	
**BMI**		
Not overweight (BMI = 16.0–24.9)	90	57.3
Overweight (BMI values ≥25.0)	69	42.3

**Table 2:** Mean rates (95%CI) of total, discretionary and healthful snacking per 10 h by context categories, with the percentage of total snacking episodes by category.

	Total Snacks		Discretionary Snacks		Healthful Snacks	
	Mean Rate (95%CI)	% of Category	Mean Rate (95%CI)	% of Category	Mean Rate (95%CI)	% of Category
Total snacking episodes	8.2 (7.4, 9.1)	100	5.2 (4.6, 5.9)	100	2.1 (1.8, 2.5)	100
**Food group**						
Confectionary	1.3 (1.1, 1.5)	15.5	1.3 (1.1, 1.5)	24.5	-	-
Snack foods	1.2 (0.9, 1.6)	14.4	1.2 (0.9, 1.6)	22.8	-	-
Cookies/cakes	1.0 (0.8, 1.4)	12.4	1.0 (0.8, 1.4)	19.6	-	-
Sugary drinks/juices	1.0 (0.8, 1.2)	12.1	1.0 (0.8, 1.2)	19.1	-	-
Fruit	0.8 (0.6, 0.9)	9.2	-	-	0.8 (0.6, 0.9)	36.0
Breads/cereals	0.8 (0.6, 1.0)	9.2	-	-	0.8 (0.6, 1.0)	35.9
Iced confectionary	0.3 (0.2, 0.4)	3.9	0.3 (0.2, 0.4)	6.1	-	-
Milk products (healthful)	0.3 (0.2, 0.6)	3.7	-	-	0.3 (0.2, 0.6)	14.5
Fast food (savoury only)	0.2 (0.1, 0.3)	2.0	0.2 (0.1, 0.3)	3.2	-	-
Vegetables	0.1 (0.1, 0.2)	1.2	-	-	0.1 (0.1, 0.2)	4.7
Processed meat	0.1 (0.0, 0.2)	1.1	0.1 (0.0, 0.2)	1.7	-	-
Savoury bakery items	0.1 (0.0, 0.1)	0.6	0.1 (0.0, 0.1)	1.0	-	-
Diet drinks	0.1 (0.0, 0.1)	0.6	0.1 (0.0, 0.1)	0.9	-	-
Nuts	0.1 (0.0, 0.2)	0.5	-	-	0.1 (0.0, 0.2)	1.8
Meat and alternatives	0.1 (0.0, 0.1)	0.4	-	-	0.1 (0.0, 0.1)	1.7
Milk products (discretionary)	0.1 (0.0, 0.1)	0.3	0.1 (0.0, 0.1)	0.4	-	-
Mixed dishes (healthful)	0.1 (0.1, 0.2)	0.2	-	-	0.1 (0.1, 0.2)	4.9
Mixed dishes (discretionary)	0.1 (0.0, 0.3)	0.1	0.1 (0.0, 0.3)	1.6	-	-
**Eating location**						
Home	3.9 (3.2, 4.5)	47.5	2.1 (1.7, 2.4)	39.5	1.2 (1.0, 1.6)	58.7
School	2.6 (2.2, 3.1)	31.7	1.6 (1.4, 2.0)	31.8	0.7 (0.5, 1.0)	33.2
Public spaces	1.8 (1.2, 2.8)	21.9	1.5 (1.0, 2.1)	28.3	0.1 (0.0, 0.6)	7.8
*-Recreation/sport*	0.2 (0.1, 0.4)	2.4	0.1 (0.1, 0.3)	2.7	0.0 (0.0, 0.0)	0.4
*-Food outlets*	0.3 (0.1, 0.7)	3.7	0.2 (0.1, 0.4)	3.2	0.0 (0.0, 0.1)	1.1
*-Retail*	0.1 (0.0, 0.2)	1.2	0.1 (0.0, 0.2)	1.0	0.0 (0.0, 0.0)	0.2
*-Other public places*	1.3 (0.8, 2.0)	15.8	1.1 (0.7, 1.7)	21.1	0.1 (0.0, 0.4)	6.0
**Snack food source**						
Home	5.6 (4.8, 6.6)	68.9	3.2 (2.7, 3.9)	62.6	1.7 (1.4, 2.1)	81.7
Food outlets	1.0 (0.6, 1.7)	12.8	0.9 (0.5, 1.4)	16.8	0.1 (0.0, 0.2)	3.0
Unknown	0.4 (0.3, 0.6)	5.0	0.3 (0.2, 0.5)	6.2	0.0 (0.0, 0.1)	1.3
School	0.4 (0.2, 0.9)	4.8	0.2 (0.1, 0.5)	3.9	0.2 (0.1, 0.5)	9.3
Other child	0.3 (0.2, 0.6)	3.8	0.2 (0.1, 0.4)	4.3	0.1 (0.0, 1.4)	2.6
Community venue	0.2 (0.0, 1.0)	2.5	0.2 (0.0, 1.0)	3.5	0.0 (0.0, 0.1)	0.5
Adult	0.1 (0.0, 0.3)	1.3	0.1 (0.0, 0.2)	1.1	0.0 (0.0, 0.3)	1.7
Retail	0.1 (0.0, 0.5)	0.8	0.1 (0.0, 4.7)	1.3	0	0
Recreation/sport	0.0, 0.0, 0.1)	0.1	0.0 (0.0, 0.1)	0.2	0	0
**Time of day**						
Morning (06:00–11.59)	2.4 (2.0, 3.0)	29.7	1.6 (1.3, 1.9)	30.9	0.6 (0.4, 0.9)	28.2
Afternoon (12:00–17.59.)	4.6 (3.9, 5.4)	55.9	2.8 (2.3, 3.5)	54.9	1.2 (1.0, 1.5)	58.2
Evening (18:00–23.59)	1.2 (0.8, 1.7)	14.0	0.7 (0.5, 1.0)	14.2	0.3 (0.1, 0.7)	13.6
**Social contact**						
None	2.0 (1.4, 2.8)	24.5	1.2 (0.1, 1.8)	22.7	0.6 (0.4, 0.9)	28.2
Children	3.6 (2.8, 4.6)	44.1	2.4 (1.8, 3.1)	46.0	0.9 (0.7, 1.8)	43.9
Adults	1.2 (1.0, 1.5)	15.1	0.8 (0.5, 1.2)	15.0	0.3 (0.2, 0.4)	12.4
Children and adults	1.3 (0.7, 2.5)	16.4	0.8 (0.5, 1.3)	16.3	0.3 (0.1, 0.7)	15.5
**Screen use**						
No screen	6.3 (5.6, 6.9)	76.0	3.9 (3.6, 4.3)	75.9	1.6 (1.3, 1.9)	74.9
Screen use	2.0 (1.5, 2.6)	24.0	1.2 (1.0, 1.6)	24.1	0.5 (0.3, 0.8)	25.1

**Table 3:** Mean rates and rate ratios (95% CI) of snacking, healthful snacking and discretionary snacking by sociodemographic characteristics and body mass index (BMI).

	All Snacks			Healthful Snacks			Discretionary Snacks		
Characteristic	Mean Rate (95%CI)	Crude Rate ratio (RR) (95%CI)	Adjusted RR (95%CI) ^1^	Mean Rate (95%CI)	Crude RR (95%CI)	Adjusted RR (95%CI) ^1^	Mean Rate (95%CI)	Crude RR (95%CI)	Adjusted RR (95%CI) ^1^
Gender									
Female (ref)	9.2 (8.2, 10.0)	1	1	2.5 (2.0, 3.0)	1	1	5.8 (5.2, 6.5)	1	1
Male	7.2 (6.4, 8.1)	**0.79 (0.68, 0.91)**	**0.80 (0.70, 0.90)**	1.7 (1.3, 2.2)	0.70 (0.49, 1.00)	**0.71 (0.55, 0.92)**	4.6 (3.8, 5.5)	**0.79 (0.65, 0.96)**	**0.80 (0.66, 0.97)**
Ethnicity									
NZE (ref)	8.5 (7.4, 9.8)	1	1	2.4 (1.9, 3.0)	1	1	5.1 (4.3, 6.1)	1	1
Māori	7.2 (5.8, 9.0)	0.85 (0.65, 1.11)	0.80 (0.63, 1.00)	1.5 (0.9, 2.5)	0.62 (0.36, 1.08)	**0.54 (0.35, 0.82)**	5.2 (4.2, 6.4)	1.01 (0.78, 1.32)	0.96 (0.74, 1.25)
Pacific	8.0 (6.8, 9.3)	0.94 (0.76, 1.15)	0.95 (0.80, 1.13)	1.4 (0.8, 2.3)	**0.57 (0.33, 0.99)**	**0.58 (0.36, 0.94)**	5.5 (4.9, 6.2)	1.08 (0.88, 1.32)	1.05 (0.88, 1.24)
BMI									
Not overweight (ref)	8.6 (7.3, 10.0)	1	1	2.3 (1.8, 2.8)	1	1	5.2 (4.3, 6.3)	1	1
Overweight/obese	7.3 (6.6, 8.4)	0.85 (0.67, 1.09)	0.87 (0.69, 1.08)	1.5 (1.2, 2.0)	**0.68 (0.49, 0.94)**	0.75 (0.53, 1.06)	5.1 (4.4, 5.9)	0.98 (0.74, 1.30)	0.95 (0.73, 1.25)
NZiDep									
Lower deprivation (ref)	7.9 (7.1, 8.8)	1	1	2.0 (1.6, 2.4)	1	1	5.0 (4.4, 5.7)	1	1
Higher deprivation	9.0 (7.0, 11.6)	1.13 (0.86, 1.49)	1.19 (0.81, 1.10)	2.5 (1.6, 4.2)	1.30 (0.73, 2.33)	1.48 (0.91, 2.43)	5.9 (4.7, 7.4)	1.18 (0.91, 1.54)	1.16 (0.85, 1.59)

Boldface indicates statistical significance. ^1^ Mutually adjusted for gender, ethnicity, BMI and NZiDep.

## Data Availability

The data presented in this study are available on request from the corresponding author. The data are not publicly available due to ethical and privacy restrictions.

## Supplementary Materials

The following are available online at https://www.mdpi.com/2072-6643/13/1/103/s1, Supplementary Material S1: Coding protocol, Supplementary Material S2: Rates of total, discretionary and healthful snacking by gender and ethnicity, delineated by eating location.
