# Supplementary material for: The Frequency and Context of Snacking among Children: An Objective Analysis Using Wearable Cameras
Source: Nutrients. 2020 Dec 30;13(1):103. doi: 10.3390/nu13010103 (PMC7824478; doi:10.3390/nu13010103)
Supplement: Supplementary file 1 [file nutrients-13-00103-s001.zip › Supplementary material 1.docx]

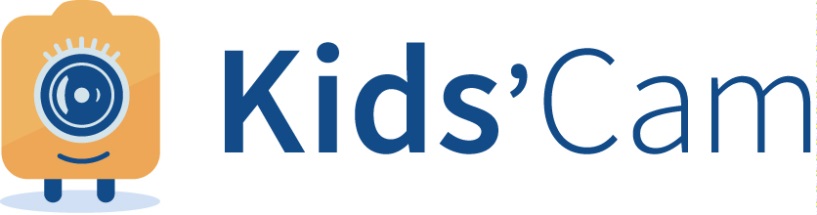


Big Snack

Coding Protocol

Contents

[Study aims 3](#_Toc51164602)

[Study Definitions 3](#_Toc51164603)

[Coding methods 3](#_Toc51164604)

[Detailed rules: eating episodes, screen use and social contact 5](#_Toc51164605)

[Coding for eating episodes 5](#_Toc51164606)

[Coding for screen use 5](#_Toc51164607)

[Coding for social contact 5](#_Toc51164608)

[Coding Examples 6](#_Toc51164609)

[Ethics 7](#_Toc51164610)

[Coding Definitions 8](#_Toc51164611)

[Setting 9](#_Toc51164612)

[Food type 12](#_Toc51164613)

[Source 14](#_Toc51164614)

[Purchaser 17](#_Toc51164615)

[Screens 18](#_Toc51164616)

[Presence of others 18](#_Toc51164617)

[Nutrient Profiling 20](#_Toc51164618)

[Core Yoghurts 20](#_Toc51164619)

[Core Breakfast Cereals 24](#_Toc51164620)

[References 34](#_Toc51164621)

# Study aims

1) To examine the frequency and context of children’s snacking behaviour.

2) To explore differences in snacking frequency by eating location (homes, schools and public spaces), sociodemographic characteristics (gender, ethnicity and household socioeconomic deprivation) and BMI.

# Study Definitions

| **Term** | **Definition** |
| --- | --- |
| Eating episode | Either a snacking episode or main meal. |
| Snacking episode | Eating episode involving consumption of an individual food/beverage or mixed meal in between main meals. |
| Main meal | Eating episode involving consumption of breakfast, lunch or dinner. |
| Mixed dish | Mixed ingredients and/or multiple items in one dish. |
| Food group | Food/beverage category e.g. confectionary, fruit. |
| Eating location | The setting where an eating episode took place e.g. school. |
| Source | The source of food/beverages consumed, e.g. home, convenience store, other children. |
| Day of week | Day that an eating episode took place (either Thursday – a school day – or Saturday – a weekend day) |
| Timing | The time that an eating episode took place, simplified in analysis stage as: morning (06:00-12:00 hours), afternoon (12:00-18:00 hours) and evening (18:00-24:00) hours. |
| Social contact | Presence of children or adults known to the participant during an eating episode. |
| Screen use | Use of screen(s) during an eating episode, such as televisions, computers and mobile devices. |
| Purchaser | The person who purchased a consumed item when evidence of purchasing was observed (participant, other child or adult) |
| Snacking prevalence | The proportion of children who snacked at least once |
| Snacking frequency | The number of snacks consumed per day. Calculated as the number of snacking episodes divided by observation time, rescaled as a rate per 10/hrs. |
| Observation time | The number of photos captured on Thursday/Saturday, multiplied by the median photo capture rate of seven seconds. |

# Coding methods

1. Enter information on each food and beverage consumed by children in the pre-formatted Excel spreadsheet (in separate rows), using the definitions for food group, product contents, eating location, source, purchasing, screen use and presence of others (Figure 1).
2. Before entering information in the spreadsheet, view the entire image sequence related to the eating episode. If you are uncertain of the product type, record the category and product columns as ‘unknown’.
   1. **Individual food items:** Enter individual food items (e.g. chocolate bar) on separate rows of the spreadsheet.
   2. **Mixed dishes with known contents:** Enter individual components of mixed dishes (e.g. bread and eggs) in separate rows of the spreadsheet, but enter the same identifying number for each component in the ‘identifier’ column. For example, eggs on toast = eggs (1 row) and bread (1 row), each with the same identifier (e.g. 1 if it is the first eating episode of the day). Coding in this way enables the ‘collapsing’ of meal components into single eating episodes, while preserving information on the types of foods consumed. Some examples of common meals/ mixed dishes with known contents include: cereal and milk (two items), toast with eggs and bacon (3 items) and a home-made smoothie with milk, strawberries and banana (3 items).
   3. **Mixed dishes with unknown contents:** If you are uncertain about the contents of a meal/mixed dish, enter ‘mixed dish’ in the food group column. Examples of mixed dishes with unknown or undistinguishable contents include curries, soups and sandwiches if the insides of the sandwich is not visible.
   4. **Condiments:** Condiments include fats and oils (e.g. margarine and butter), sugar, salt, spreads and sauces. These are often added to individual food items or meals. Enter information on condiments in the ‘Condiments’ column (in the row of the food item it is consumed with). If a child eats a condiment on its own (e.g. they drink tomato sauce), enter the condiment in its own row.
3. A snacking episode is considered ‘complete’ when five minutes (approximately 43 images) pass without further evidence of consumption. After this time, further snacking on the same item is considered a new snacking episode. You can determine the amount of time that has past by viewing the image time stamps.
4. If you are unsure which code to use, enter ‘Check’ in that column. The eating episode will then be reviewed by the research team (see Reliability testing below).
5. You **MUST** take a short break every 30 minutes of annotation as continuous spells of annotation over this time are prone to measurement error.

Figure 1. Example of the coding spreadsheet (some columns e.g. timing not shown)

| **P_num** | **Eating episode** | **setting** | **Food type** | **Product** | **Source** | **Purchased by** | **screens** | **Presence of others** | **condiments** |
| --- | --- | --- | --- | --- | --- | --- | --- | --- | --- |
| 1001001 | Snack | school | Fruit | Banana | Home | Child | none | Children | none |
| 1001001 | Snack | school | sugary drinks | soft drink | Other child | n/a | none | children | none |
| 1001001 | Dinner | home | mixed meal | unknown | Home | n/a | TV | Adults | none |
| 1201302 | Snack | street | fast food | french fries | Fast food outlet | Fast food | none | No | none |

## Detailed rules: eating episodes, screen use and social contact

### Coding for eating episodes

You may code for an eating episode when one of two conditions were met: 1) The image sequence shows participants lifting a food or beverage towards their face; or 2) The image sequence shows participants handling a food or beverage, accompanied by evidence that the product is decreasing in quantity

Decide on the eating episode (meal vs. snack) based on the time of image capture and, if appropriate, other contextual information observed in image sequence. The main criteria for coding a meal is timing, with time windows as follows: breakfast (06:00- 09:00), lunch (11.00- 15.00) and dinner (17:00- 20:00). Breakfast is usually the first meal of the day. If children sleep past the time window, you may code the first meal of the day as breakfast. Lunch is generally easy to ascertain at school: it often takes place at the same time. Some schools also require children to eat lunch indoors before going outside.

In some cases, you may code food eaten during a meal window as a snack if it clearly was not part of a main meal. To meet this criteria, the food consumed must have been different than that eaten during the meal AND eaten in a different context than the meal (e.g. in a different setting). Examples include:

1. Children eating chocolate while traveling to school (i.e. after eating breakfast)
2. Children eating candy 30mins before lunch.
3. Children eating fruit 30mins after dinner.

### Coding for screen use

To code for screen use, there must be evidence that the participant interacted with a screen for most of the eating episode (>50% of the time). This should exclude brief glances at mobile devices.

### Coding for social contact

To code for social contact (i.e. presence of other people), there must be evidence that the person in question is known to the participant. Additionally, the person must have been present for most of the eating episode (>50% of the time). Review image sequences before and after the episode to help identify whether the person is known to the participant, as friends/family are often seen throughout the day. Examples of social contact include:

1. Eating with family at home, e.g. on a couch watching television.
2. Eating while walking with friends after school.
3. Eating lunch at school in presence of other children (note: it is likely that most eating episodes in school will involve social contact with other children).

## Coding Examples


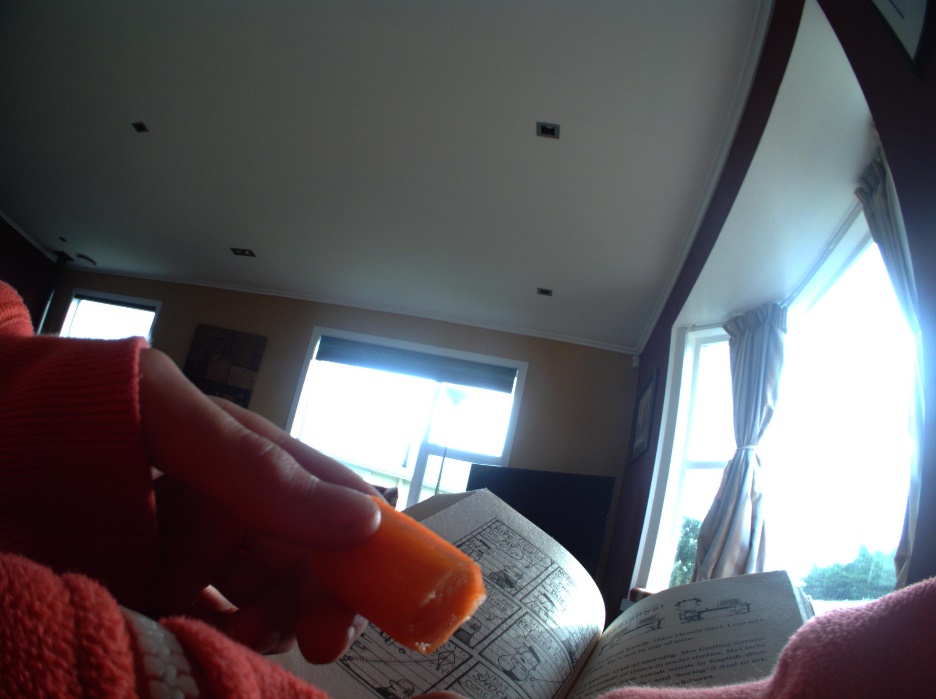


Eating episode: **Snack** 🡪 Time: 153408 🡪 Setting: **Home** 🡪 Food type: **Vegetables** 🡪 Product/contents: **Carrot** 🡪 Source: **Home**  🡪 Purchaser: **N/A** 🡪 Screen(s) **no** 🡪 Presence of others: **No**


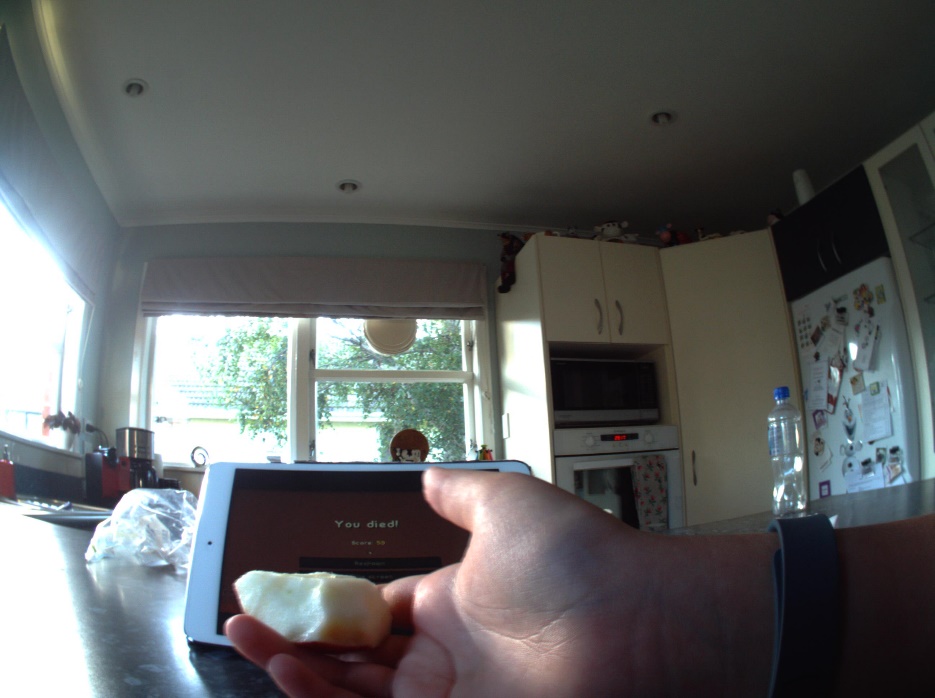


Eating episode: **Snack** 🡪 Time: 103911 🡪 Setting: **Home** 🡪 Food type: **Fruit** 🡪 Product/contents: **Apple** 🡪 Source: **Home**  🡪 Purchaser: **N/A** 🡪 Screen(s) **Computer** 🡪 Presence of others: **Yes (but not shown in this image).**

## Ethics

1. Keep the identifiable features of the data **confidential;** these features of the data should not be discussed with anyone outside the research team.
2. Do not leave data or equipment containing unsecured data unattended. If you leave your computer for any amount of time you must **log out.**
3. The University of Otago (Wellington) possesses ownership of all image data. Applicants cannot copy data without the written approval of the Principal Investigator or retain copies of the data after completion of work. Any data copied or released must be stored on a password protected device and must have gone through the appropriate anonymised procedure.
4. Protect the anonymity of all participants, third parties and their environments. To protect the privacy of those who may be inadvertently captured in the images, all images used in disseminated material will have identifiable people, street names, places, retail outlets, businesses and school names blurred. The demographic information collected will only be viewed by the core Kids’Cam team.

## Coding Definitions

**Eating Episode**

Definition: What meal of the day is it? Breakfast, lunch, dinner or a snack?

| Breakfast | First meal of the day, **usually** between the time of waking up and 9am. |
| --- | --- |
| Lunch | Meal usually from 11am to 3pm, or during school lunch breaks eg. Sandwich, pie etc. |
| Dinner | Last meal of the day, usually from 5pm to 8pm eg. mixed dishes. |
| Snack - event | Any individual food item (or meal) consumed outside the three main meals. |
| Unsure | Coder is not 100% sure what the eating episode is. |

### Setting

Definition: The place or type of surroundings where the eating event took place

Note: If a food item is consumed across multiple settings (e.g. starting in car and ending at home), only enter the setting that the child spent the most time in.

| **Setting** | **Definition** |
| --- | --- |
| Home | Includes all spaces within the home gates and boundaries i.e. indoor and outdoor spaces; or someone else’s home.  The place where one lives permanently, especially as a member of a family or household (Oxford Dictionaries, 2015). |
| School | Indicated by the presence of classroom features such as desks, tables and chairs, other children, teaching staff, school buildings and playgrounds.  School grounds are delineated by a gate and/or fence.  School is an institution for educating children and includes the building used by the school (Oxford Dictionaries, 2015). |
| Street | Roads, footpath and courtyards.  The roads or public areas of a city or town (Oxford Dictionaries, 2015). |
| Bakery | Inside an independent store selling fresh baked goods, e.g. Jack’s bakery, Baker’s Delight  A place where bread and cakes are made or sold (Oxford Dictionaries, 2015). |
| Community venue | Library - A building or room containing [collections](http://www.oxforddictionaries.com/definition/english/collection#collection__9) of books, [periodicals](http://www.oxforddictionaries.com/definition/english/periodical#periodical__2), and sometimes [films](http://www.oxforddictionaries.com/definition/english/film#film__10) and recorded music for use or [borrowing](http://www.oxforddictionaries.com/definition/english/borrowing#borrowing__2) by the public or the members of an institution (Oxford Dictionaries, 2015).  Recreation centre/community hall - a public space where meetings are held  Marae - includes the meeting house, dining hall, education and associated facilities and residential accommodation associated with the Marae.  Church - A building used for public Christian [worship](http://www.oxforddictionaries.com/definition/english/worship#worship__2) (Oxford Dictionaries, 2015). |
| Convenience store | A smaller style food retail store with two or fewer checkouts (Thornton & Kavanagh, 2012). E.g. dairy, Fix, Seven Eleven, neighbourhood corner store; Four Square; Does not include tuck shop.  When the number of checkouts is not clearly visible then look for identifiable features such as independent store names (e.g. David’s food market) or the words **Dairy, Convenience Store, Fix** etc. |
| Fast food | Includes all major fast food franchised chain restaurants e.g. McDonald’s; KFC; Burger King; Pizza Hut; Dominos; Subway; Hell Pizza; Dominos.  Easily [prepared](http://www.oxforddictionaries.com/definition/english/prepare#prepare__9) [processed](http://www.oxforddictionaries.com/definition/english/process#process-2__2) food served in [snack bars](http://www.oxforddictionaries.com/definition/english/snack-bar#snack-bar__2) and restaurants as a quick [meal](http://www.oxforddictionaries.com/definition/english/meal#meal-2__2) or to be [taken away](http://www.oxforddictionaries.com/definition/english/take#take__115) –Oxford dictionaries  Smaller takeaway food outlets such as roast chicken, Asian/Indian takeaways, pizza, and fish and chip stores or cafes where food is purchased for home consumption. (Thornton & Kavanagh, 2012). |
| Full service restaurant | Restaurant or café setting with table service, wait staff (Powell & Nguyen, 2013). |
| Fresh food market | Characterized by being outdoor, primarily selling fresh fruit, vegetables, fish and other perishables food products.  These may be large or small local markets. May only be open a few days a week (Thornton & Kavanagh, 2012) |
| Other retail | General product retailers including K-Mart, The Warehouse, Mitre 10, Bunnings; also Whitcoulls, and game and video stores.  Primary purpose is something other than food retail or the sale of petrol |
| Outdoor recreation space | Parks - A large public [garden](http://www.oxforddictionaries.com/definition/english/garden#garden__5) or area of land used for [recreation](http://www.oxforddictionaries.com/definition/english/recreation#recreation__2) (Oxford Dictionaries, 2015). Characterized by the presence of large open grassed spaces possibly with some equipment such as climbing frames or playgrounds (not primarily used for organised sport).  Walking track - A rough path or road, typically one beaten by use rather than [constructed](http://www.oxforddictionaries.com/definition/english/construct#construct__2) (Oxford Dictionaries, 2015). Characterized by in-bush or off-road areas such as the town belt.  Beach- A [pebbly](http://www.oxforddictionaries.com/definition/english/pebble#pebble__2) or [sandy](http://www.oxforddictionaries.com/definition/english/sandy#sandy__2) shore, especially by the sea between high- and low-water marks (Oxford Dictionaries, 2015).  River - A large natural stream of water flowing in a [channel](http://www.oxforddictionaries.com/definition/english/channel#channel__2) to the sea, a lake, or another river (Oxford Dictionaries, 2015) |
| Private transport | Inside a car, van or truck |
| Public transport - facility | Associated with public transport facilities – e.g. bus shelters, train stations, airports etc. |
| Public transport - vehicle | Inside a bus, train, airplane, ferry |
| Service station – on-site | An [establishment](http://www.oxforddictionaries.com/definition/english/establishment#establishment__2) beside a road [selling](http://www.oxforddictionaries.com/definition/english/sell#sell__3) [petrol](http://www.oxforddictionaries.com/definition/english/petrol#petrol__2) and oil and sometimes having the facilities to [carry out](http://www.oxforddictionaries.com/definition/english/carry#carry__74) [maintenance](http://www.oxforddictionaries.com/definition/english/maintenance#maintenance__2) (Oxford Dictionaries, 2015)  An establishment selling petrol and food including Z, Caltex, BP, Mobil. This definition includes the petrol pumps and forecourt area but not the street-side advertisements (coded as street). |
| Shopping mall | A large enclosed indoor shopping area from which traffic is excluded (Oxford Dictionaries, 2015).  Includes food courts |
| Sport | Swimming pool - council facility/publically accessible swimming pool  Indoor sports stadium - sports stadiums that are used for recreational sporting games e.g. ASB stadium  Outdoor sports stadium - large regional stadiums where professional matches are held e.g. Westpac Stadium  Sports clubrooms - club emblems and colours are on display  Sports ground - outdoor area designed primarily for the purpose of playing sport (buildings and other associated structures) |
| Supermarket | Inside a supermarket with three or more checkouts. Sells fresh fruit and vegetables. Has long opening hours. (Thornton & Kavanagh, 2012). E.g. Countdown, Pac’n’Save, New World, Moore Wilsons. |

### Food type

Category that the consumed food item belongs to. Note: use the diagrams on pages 20-32 to distinguish between healthy and unhealthy milk products and cereals. If a milk or cereal product is not pictured in these diagrams, it is an unhealthy product.

| **Category** | **Definitions** |
| --- | --- |
| Breads and cereals | All breads and cereals, rice, pasta, noodles, crackers; rice crackers; flat breads; crumpets; instant noodles. Excludes cereals with >15g/100g total sugars; see nutrient profiling document for exemptions |
| **Cereal (unhealthy)** | Cereals with > 15g /100g total sugars; see nutrient profiling document for exemptions |
| Cereal (unclear) | Use when core vs. noncore is not clear (e.g. blocked label) |
| Confectionary | Chocolate and confectionary, chocolate coated products |
| Cookies and cakes | Cakes, muffins, sweet biscuits, sweet pies, sweet pastries, slices |
| Diet drinks | Artificially sweetened beverages – e.g. Diet Coke, Pepsi Max, V Sugar-free, PowerAde Zero (excludes reduced sugar drinks e.g. Coke Life) |
| Fast food | Includes food and meals that are designed for ready availability, use, or consumption and sold at eating establishments for quick availability or take-out; McDonalds, Burger King, KFC, Dominos, Pizza Hut, fish and chips, other takeaway food; Subway; Pita Pit; Wishbone. |
| Fruit | Includes dried fruit, canned, fresh and frozen |
| Ice cream | Ice cream and iced confectionary |
| Meat and alternatives | Meat/eggs/nuts - fresh meat, nut products such as peanut butter; legumes; fish (Note: Processed meats are **excluded** from this category) |
| Milk products (healthy) | Plain milk; cheese; yoghurt; milk alternatives – soy, rice, almond. Includes milk products <10g/100g sugar; see nutrient profiling document for exemptions |
| Milk products (unhealthy) | Dairy food and yoghurt >10g/100g total sugars, custard; see nutrient profiling document for exemptions |
| Milk products (unhealthy) | Use when core vs. noncore is not clear (e.g. blocked label) |
| Mixed meals | Sandwiches; sushi; Kabab; burger (homemade); |
| Savoury bakery items | Pies, scones, sausage rolls |
| Processed meats | Salami, sausages, beef jerky and dried meats, bacon, ham, delicatessen meats; chicken nuggets |
| Snack bars | Muesli bars, fruits straps, fruit squeezies |
| Snack foods | Potato chips, popcorn, corn chips, extruded snacks |
| Sugary drinks and juices | Includes carbonated beverages and soft drinks including Coke Life; sports drinks; energy drinks; flavoured milks (chocolate milk); fruit drinks (e2, Ribena); powdered drinks (Milo, Nesquik, Raro); cordial; fruit juices; iced tea; breakfast drinks (UP&GO); flavoured waters |
| Vegetables | Includes dried fruit, canned, fresh and frozen |
| Water | Plain |
| Undetermined | Coder is not 100% certain of the food item/product |

### Source

Definition: Where the participant or child gets the food product from.

| Home | If the food item has been packed from home. For example - in a lunch box or school bag.  Food products that a participant consumes at home, where there is no evidence in previous image sequences that the source is from elsewhere (anywhere that is not ‘home’).  You can assume that ‘home’ is the source for foods in a child’s lunch box/ school bag (e.g. chips and glad-wrapped sandwiches). |
| --- | --- |
| Convenience store | Product purchased from convenience store. |
| Supermarket | Food purchased from a supermarket. |
| School stall/ canteen | Food from a stall/canteen within the school grounds |
| Street stall | A stall on the side of the street |
| Child | Obtained from a peer or any other child |
| Adult | Obtained from an adult companion e.g. parent, teacher etc |
| Mobile food vendor | A food stall contained on a motor vehicle or that is designed to be moved by a motor vehicle (e.g. a food stall in caravan or on a trailer) (Auckland City Council, 2013).  Includes food truck selling food such as ice cream, fast food, typically at a market, sports or cultural event. |
| Market | Characterized by being outdoor, primarily selling already cooked food and other product goods that is not food.  These may be large or small local markets. May only be open a few days a week (Thornton & Kavanagh, 2012) |
| Fast food restaurant | Smaller takeaway food outlets or cafes where food is purchased to eat in or for home consumption. (Thornton &amp; Kavanagh, 2012).  Easily prepared processed food served in snack bars and restaurants as a quick meal or to be taken away –Oxford dictionaries |
| Full service restaurant | Restaurant or café setting with table service, wait staff (Powell &amp; Nguyen, 2013). |
| Vending machine | An electronic machine used to dispense a food product after money has been put in the machine. |
| Unknown | Coder is not 100% certain what the source is. |

### Purchaser

Definition: Who has completed the product purchase?

(Only applicable if the source is either ‘convenience store, supermarket, school stall, street stall, market or mobile food vendor’) i.e. If the source is supermarket, then who made the purchase? The participant, other child or other adult? However, if the source is home then this category is marked N/A for not applicable.

| Own purchase | The participant has made the product purchase |
| --- | --- |
| Other child | A child other than the participant has completed the product purchase |
| Other adult | An adult has completed the product purchase |
| N/A (not applicable) | If the photo sequences for a participant does not show a food purchase prior to an eating episode, then the ‘purchaser’ category is marked as N/A. |
| Unidentified | Coder is not 100% certain who made the food product purchase |

### Screens

Interaction with screens during the eating episode.

| Phone | Smartphones, cellphones, etc. |
| --- | --- |
| Computer | Laptops, desktops, etc. |
| Television | An adult has completed the product purchase |
| Multiple | Multiple screens, e.g. smartphone and TV. |
| Unidentified | Coder is not 100% certain whether the participant interacted with a screen. |

### Presence of others

Presence of other people known to the participant, e.g. friends and family. (Children snack more when in the presence of others).

| Children | E.g. friends at convenience store |
| --- | --- |
| Adults | E.g. parents at restaurant |
| Children and adults | E.g. family at dinner table |
| Unidentified | Coder is not 100% certain whether the participant interacted with a screen. |

## Nutrient Profiling

### Healthy Yoghurts

| 1. Meadow Fresh Yoghurt Smoothie **Products Range** | 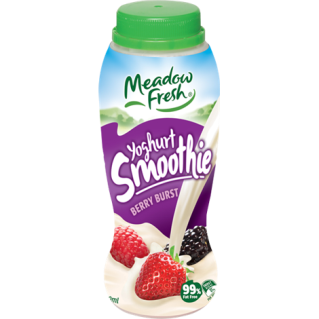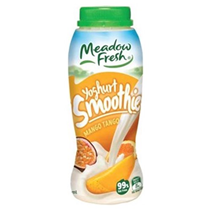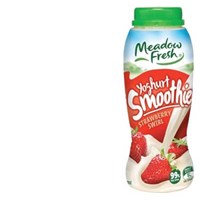 |
| --- | --- |
| 1. Meadow Fresh Lite Yoghurt **Product Range 1kg** | 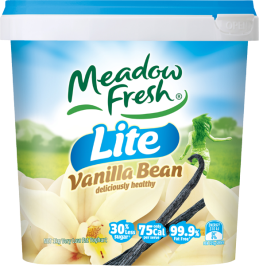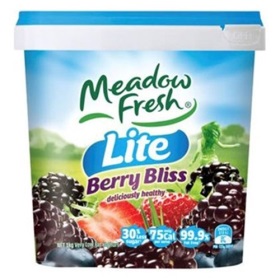 Examples |
| 1. Meadow Fresh Natural Yoghurt | 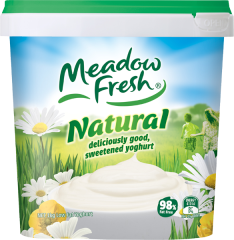 |
| 1. Anchor Greek Style Yoghurt **Product Range** | 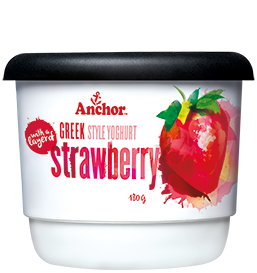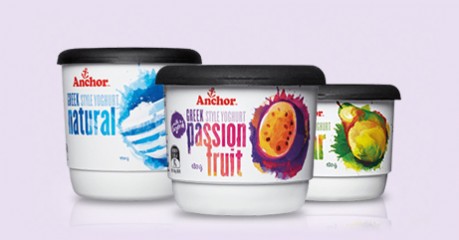 |
| 1. Anchor Uno Strawberry | 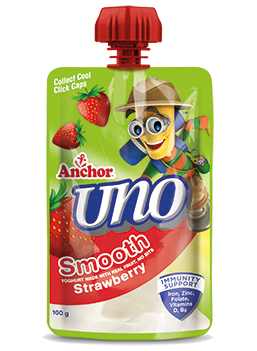 |
| 1. CalciYum Original Chocolate Dairy food | 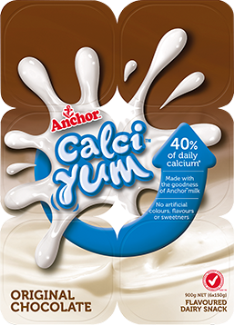 |
| 1. CalciYum Wicked Chocolate Dairy Food | 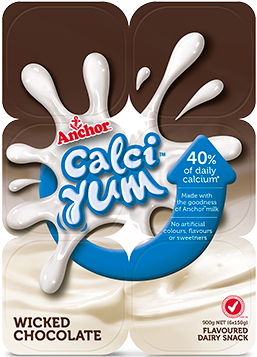 |
| 1. Fresh n' Fruity Yoghurt Lite **Product Range** | 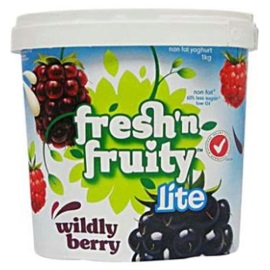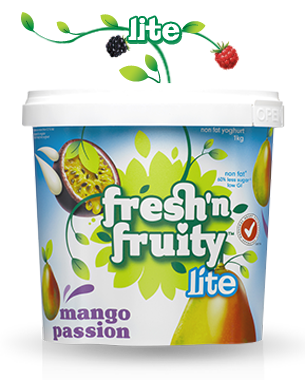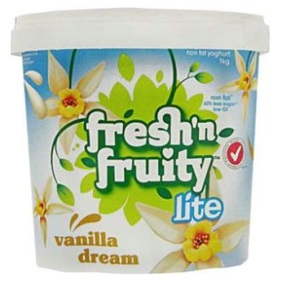 Examples |
| 1. Fresh n' Fruity Simply Strawberry | 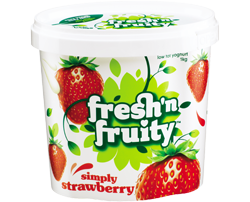 |
| 1. Fresh n' Fruity Simply Apricot | 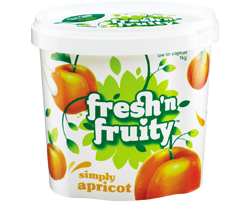 |
| 1. The Collective Kefir Unsweetened Pourable Yoghurt | 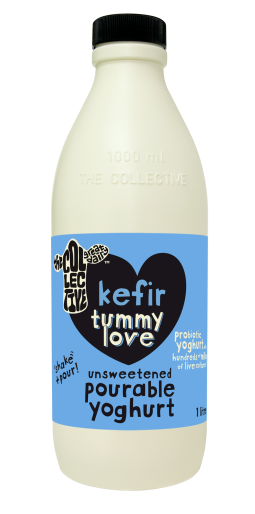 |
| 1. Organic Cyclops Yoghurt Low Fat | 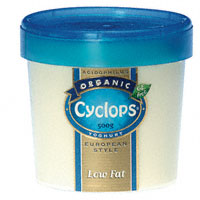 |
| 1. Organic Cyclops Yoghurt Strawberry | 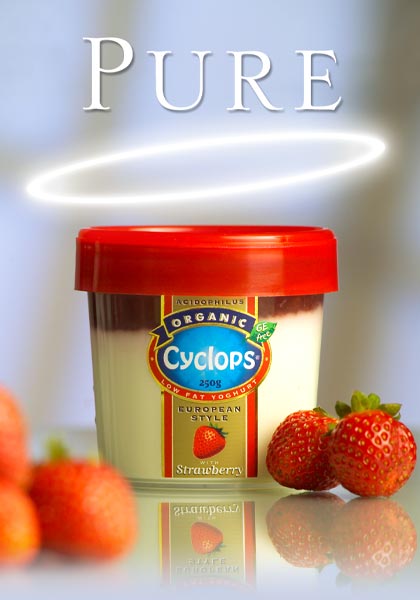 |
| 1. Organic Cyclops Yoghurt Boysenberry | 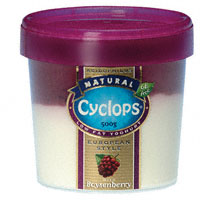 |
| 1. Organic Cyclops Yoghurt Raspberry | 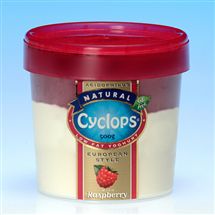 |
| 1. Organic Cyclops Yoghurt Banana |  |
| 1. Organic Cyclops Yoghurt Low fat Greek | 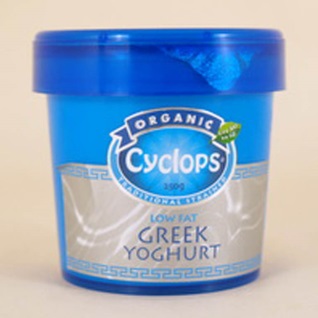 |
| 1. Symbio Yoghurts – **Whole Product Range** | 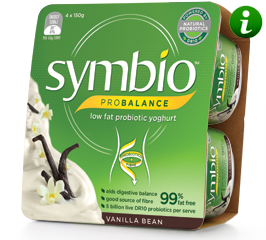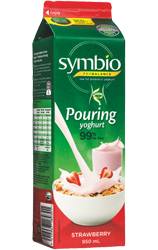 |
| 1. Yoplait Yoghurt Delite- Mixed Berry | 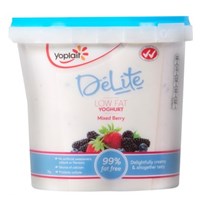 |
| 1. Yoplait Yoghurt Delite- Peach & Mango | 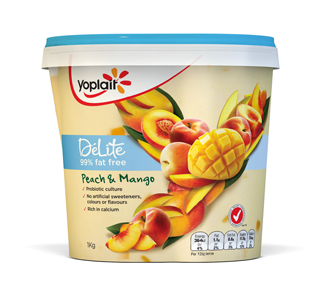 |
| 1. Yoplait Yoghurt Elivaé **– Whole Product Range** | 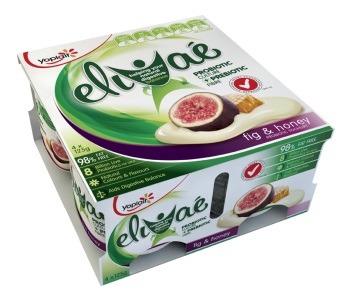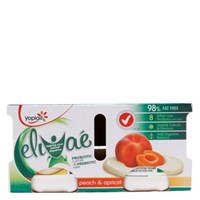 |
| 1. Yoplait Yoplus Trim | 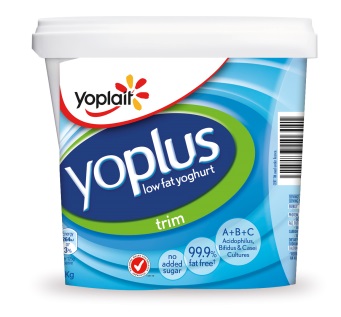 |
| 1. Yoplait Yogo Xtreme Choc | 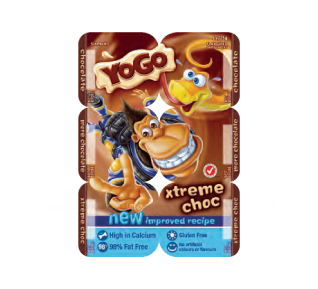 |
| 1. DeWinkel Natural Plain Unsweetened Yoghurt | 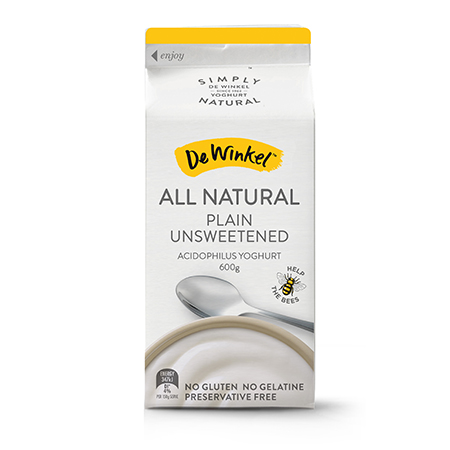 |
| 1. Naturalea Natural Plain Unsweetened Yoghurt | 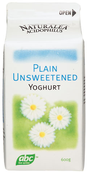 |

### Healthy Breakfast Cereals

| Sanitarium Weet-Bix | 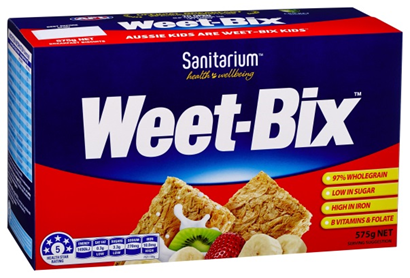 | Sanitarium Skippy Cornflakes | 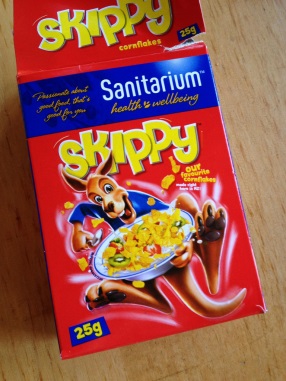 |
| --- | --- | --- | --- |
| Sanitarium Weet-Bix Gluten Free | 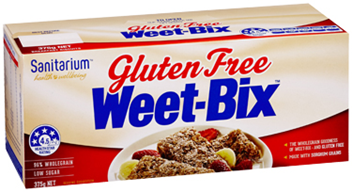 | Sanitarium Ricies | 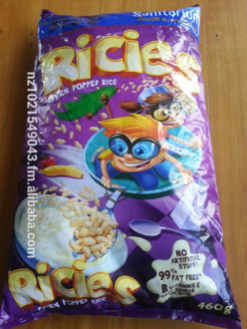 |
| Sanitarium Weet-Bix Multigrain | 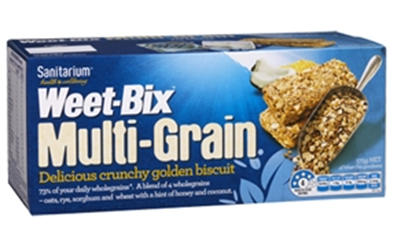 | Sanitarium Weeties | 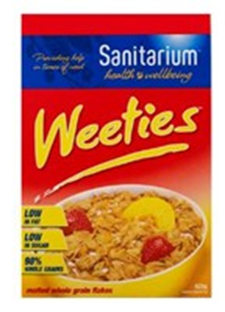 |
| Sanitarium Weet-Bix Hi Bran | 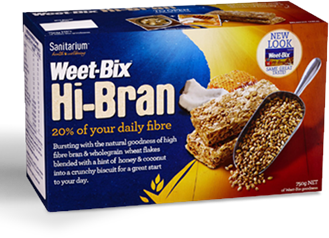 | Sanitarium Fibre Life Bran Flakes | 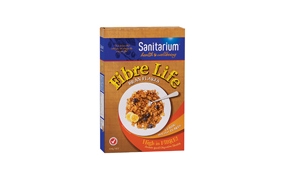 |
| Sanitarium Weet-Bix Oat Bran | 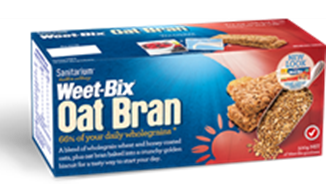 | Sanitarium Puffed Wheat | 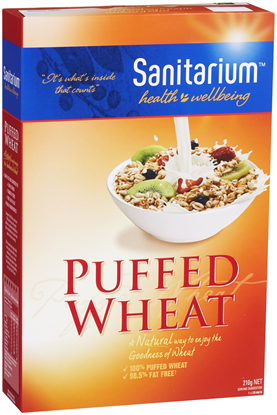 |
| Sanitarium Bran | 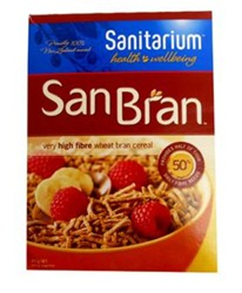 | Hubbards Thank Goodness Gluten-Free Rice Pops | 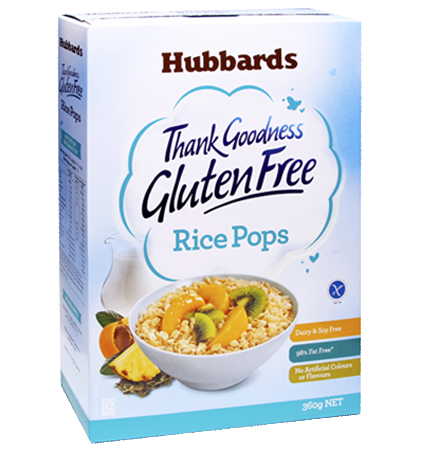 |
| Hubbards Simply Natural Muesli Berry | 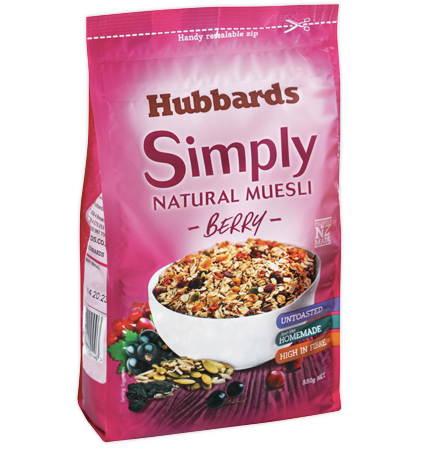 | Hubbards Thank Goodness Gluten-Free Brown Rice Porridge Maple | 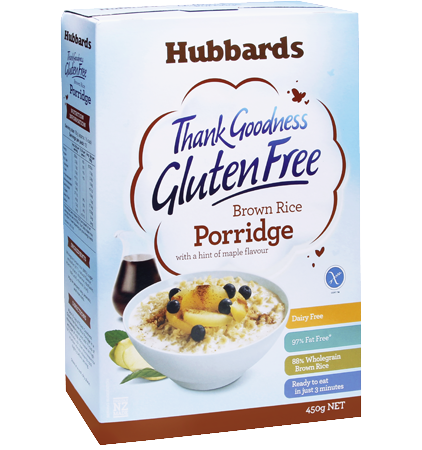 |
| Hubbards Lite & Right Hazelnut and Almond | 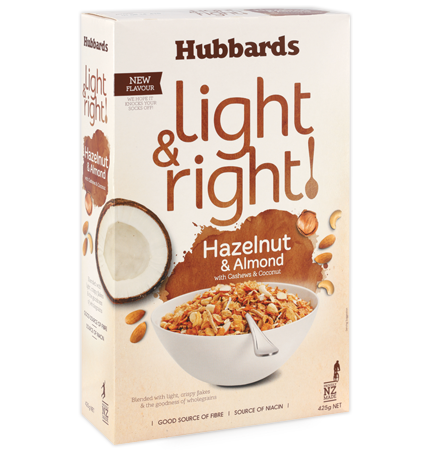 | Hubbards Bran Nuts | 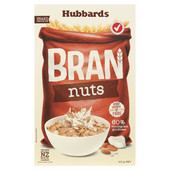 |
| Hubbards Thank Goodness Gluten-Free Cornflakes | 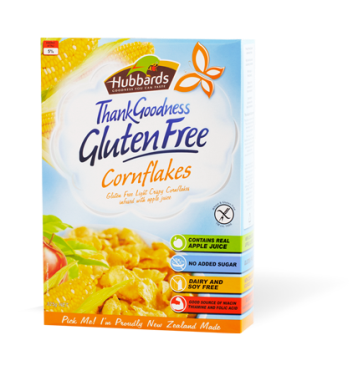 | Kellogg’s All Bran Wheat Flakes | 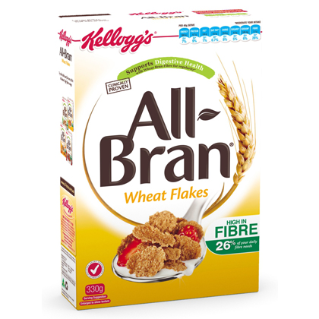 |
| Kellogg’s Special K - Original | 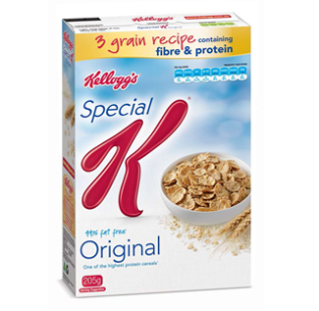 | Kellogg’s® Five Whole Grain Muesli – Roasted Almonds, Coconut & Flame Raisins | 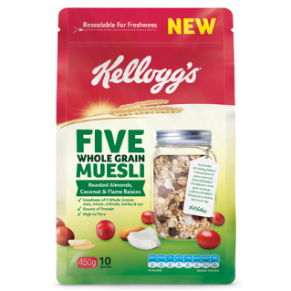 |
| Kellogg’s Corn Flakes | 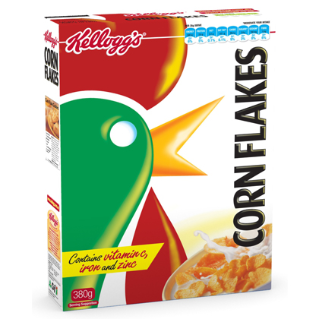 | Vogel's Original Muesli - Natural Apricot | 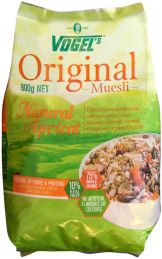 |
| Kellogg's Rice Bubbles | 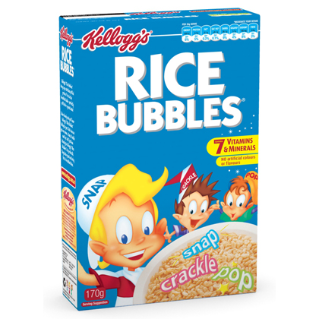 | Harraways Rolled Oats | 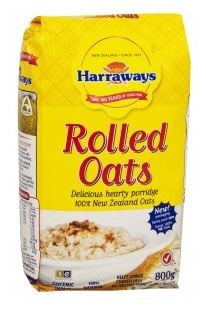 |
| Kellogg's Gaurdian | 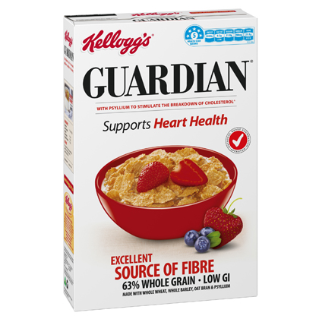 | Harraways Scotch Oats | 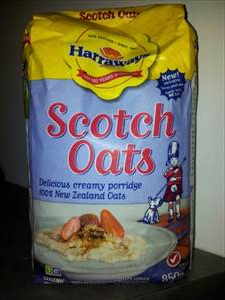 |
| Kellogg's Mini-Wheats Little Bites | 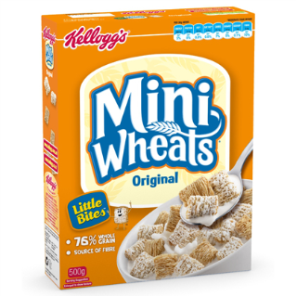 | Harraways Fruit Harvest | 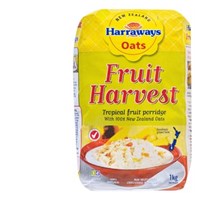 |
| Harraways Organic Rolled Oats | 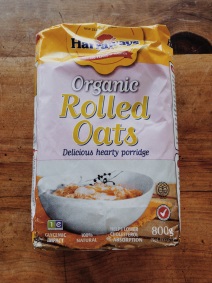 | Harraways Wholegrain Oats | 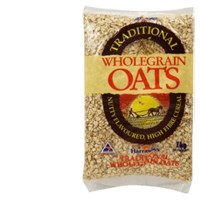 |
| Harraways Organic Wholegrain Oats | 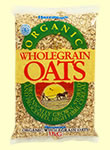 | Uncle Toby's Traditional Oats | 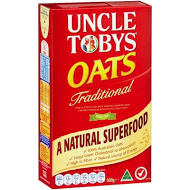 |
| Harraways Oat singles - Plain | 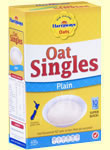 | Uncle Toby's Quick Oats | 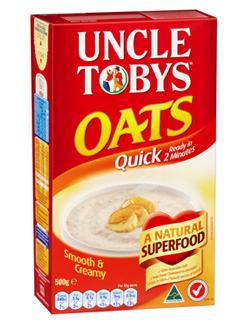 |
| Harraways Muesli - Original | 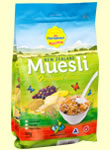 | Uncle Toby's Oats Quick Sachets - original | 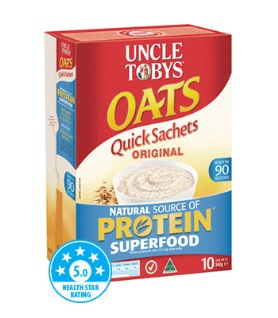 |
| Nicolas Organic Oat singles - Plain | 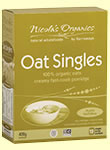 | Uncle Toby's Cheerios Wholegrain | 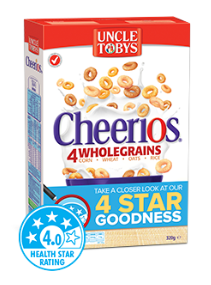 |
| Ceres Organics Organic Rolled Oats, Jumbo Wholegrain | 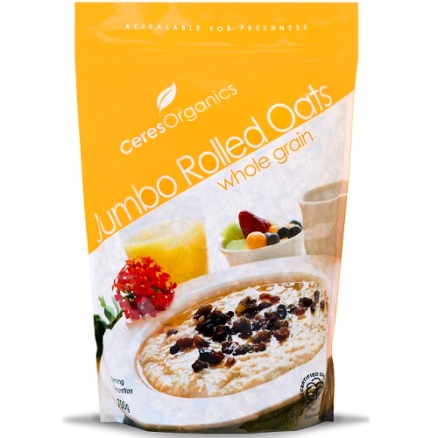 | Ceres Organics Organic Cereal, Quinoa | 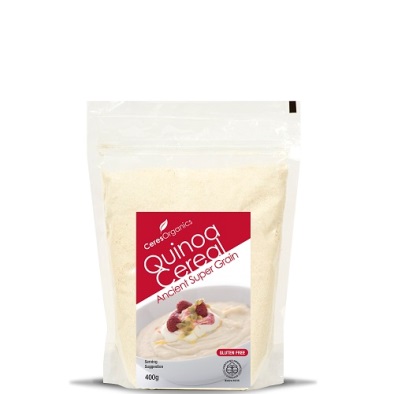 |
| Ceres Organics Organic Amaranth Flakes | 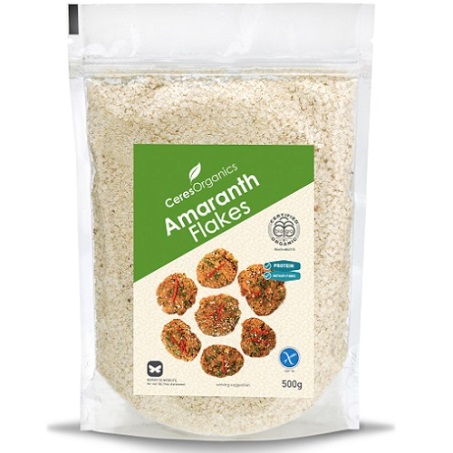 | Ceres Organics Organic Cereal, Millet | 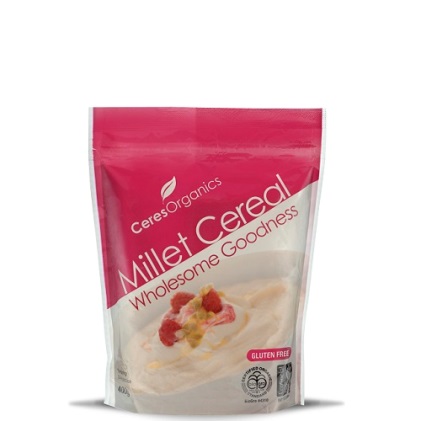 |
| Ceres Organics Organic Amaranth Puffs | 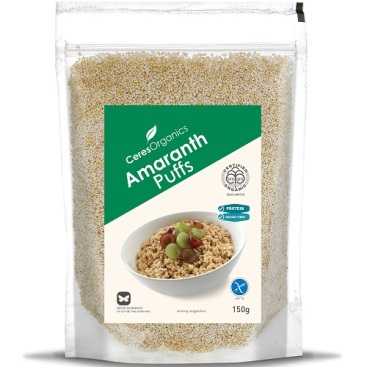 | Ceres Organics Organic Cereal, Rice | 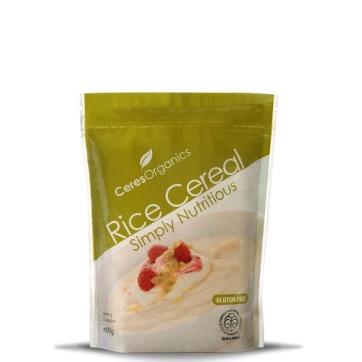 |
| Ceres Organics Organic Bircher Muesli - Original | 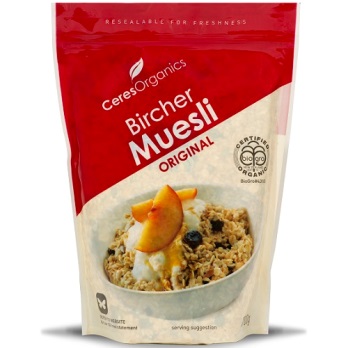 | Ceres Organics Organic Hot Cereal - Quinoa, Cacao & Millet | 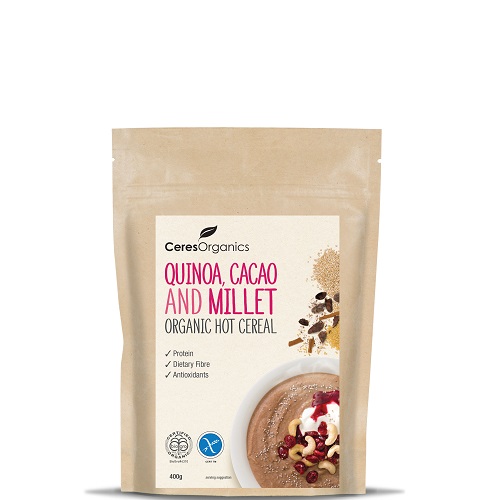 |
| Ceres Organics Organic Buckwheat Cereal | 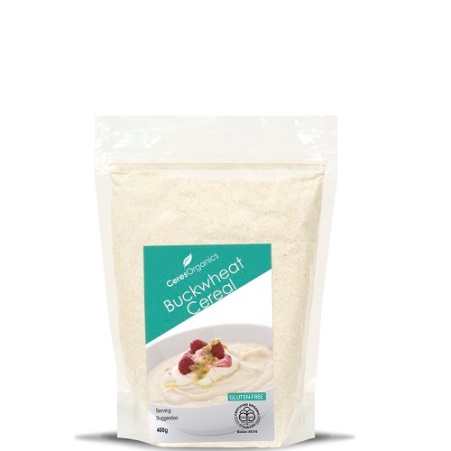 | Ceres Organics Organic Oat Bran | 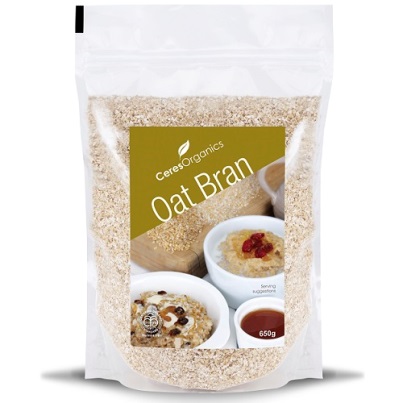 |
| Ceres Organics ORGANIC QUINOA FLAKES | 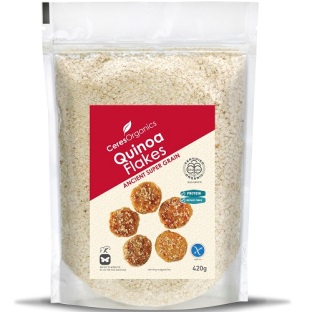 | Freedom Foods Active Balance Buckwheat & Quinoa | 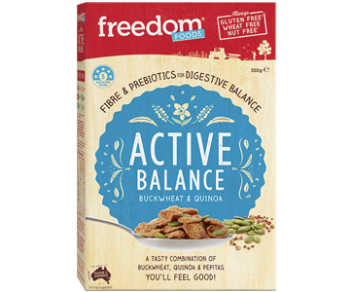 |
| Ceres Organics ORGANIC QUINOA PUFFS | 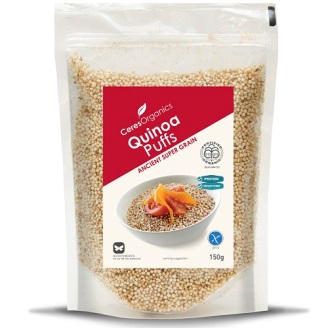 | Freedom Foods Ancient Grain Flakes | 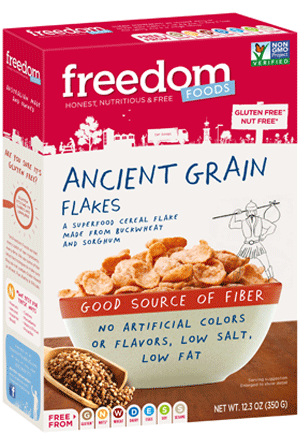 |
| Ceres Organics ORGANIC ROLLED OATS, WHOLEGRAIN QUICK COOK | 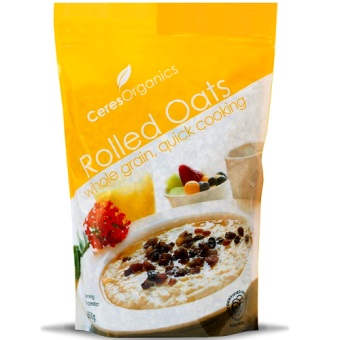 | Freedom Foods Corn Flakes | 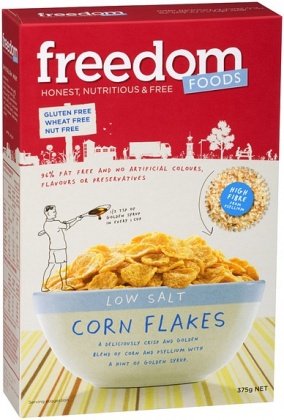 |
| Ceres Organics ORGANIC STEEL CUT OATS | 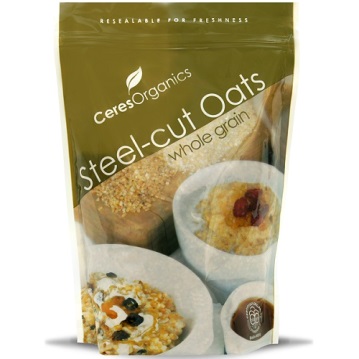 | Freedom Foods Rice Puffs | 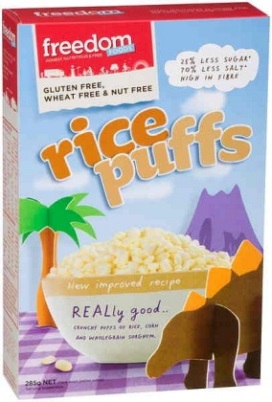 |
| Freedom Foods Active Balance Multigrain & Cranberry | 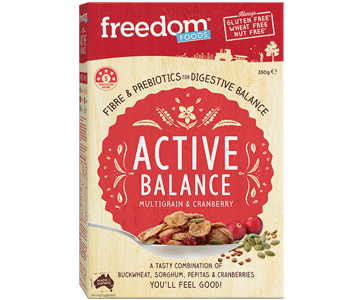 | Freedom Foods Rice Flakes | 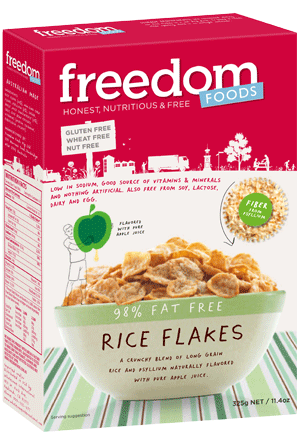 |
| Freedom Foods Muesli Ancient Grains | 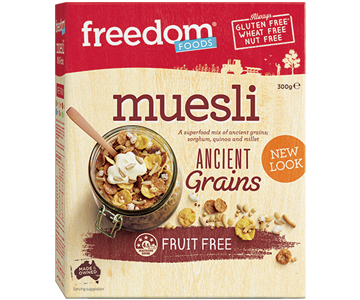 | Freedom Foods Porridge | 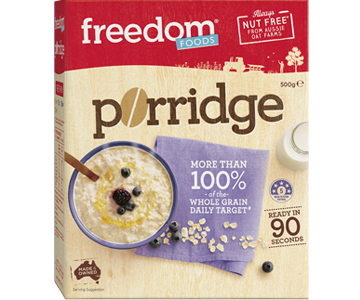 |
| Healtheries Simple Wheat & Gluten Free – Wholegrain Hot Cereal | 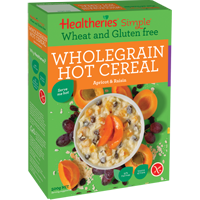 | Natures Path Corn Flakes | 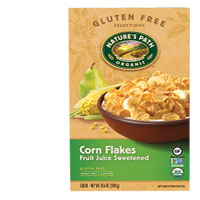 |
| Natures Path Corn Puffs | 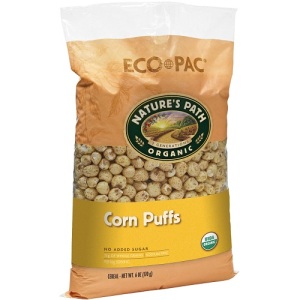 | Natures Path Heritage Flakes | 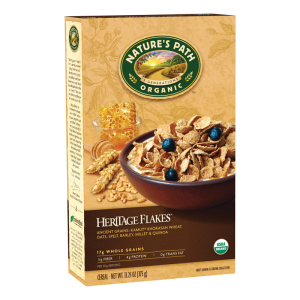 |
| Natures Path Crispy Rice | 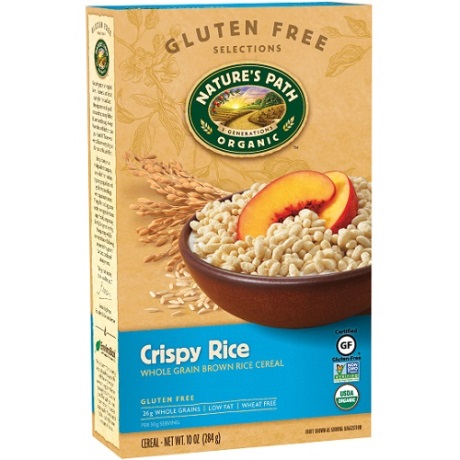 | Natures Path Mesa Sunrise Flakes |  |
| Natures Path Honey'd Corn Flakes |  | Natures Path Millet Puffs |  |
| Natures Path Kamut puffs |  | Natures Path Millet Rice flakes |  |
| Natures Path Rice Puffs |  |  |  |

## References

Auckland City Council. (2013). Food Safety Bylaw 2013 Auckland Auckland City Council

Hutt City Council. (2014). Definitions *Hutt City Council Distrcit Plan 2004*

(Vol. Updated 14 November 2014, pp. 12).

Kelly, B., & Chapman, K. (2007). Food references and marketing to children in Australian magazines: A content analysis. *Health Promotion International,* ***22***(4), 284-291.

Kelly, B., King, L., Jamiyan, B., Chimedtseren, N., Bold, B., Medina, V. M., De los Reyes, S. J., Marquez, N. V., Rome, A. C. P., Cabanes, A. M. O., Go, J. J., Bayandorj, T., Carlos, M. C. B., & Cherian Varghese, C. (2015). Density of outdoor food and beverage advertising around schools in Ulaanbaatar (Mongolia) and Manila (The Philippines) and implications for policy. *Critical Public Health,* ***25***(3), 280-290.

Oxford Dictionaries. (2015). Dictionary Retrieved 27/10/15

Thornton, L. E., & Kavanagh, A. M. (2012). Association between fast food purchasing and the local food environment. *Nutrition & diabetes,* ***2***(12), e53.

World Health Organization. (2012). A framework for implementing the set of reccomendations on the marketing of foods and non-alcoholic beverages to children. Switzerland: World Health Organization.

World Health Organization. (2015). *WHO Regional Office for Europe nutrient profile model*. Copenhagen, Denmark: World Health Organization
