# Supplementary material for: The Frequency and Context of Snacking among Children: An Objective Analysis Using Wearable Cameras
Source: Nutrients. 2020 Dec 30;13(1):103. doi: 10.3390/nu13010103 (PMC7824478; doi:10.3390/nu13010103)
Supplement: Supplementary file 1 [file nutrients-13-00103-s001.zip › Supplementary material 2.docx]

# Supplementary material 2

Supplementary table 1. Mean rates and rate ratios (95%CI) of snacking, healthful snacking and discretionary snacking by gender and eating location

| Eating location | Gender | All snacks | | Discretionary snacks | | Healthful snacks | |
| --- | --- | --- | --- | --- | --- | --- | --- |
|  |  | Mean rate (95%CI) | Adjusted RR (95%CI) | Mean rate (95%CI) | Adjusted RR (95%CI) | Mean rate (95%CI) | Adjusted RR (95%CI) |
| Homes | Female | 3.7 (3.3, 4.3) | 1 (ref) | 1.9 (1.6, 2.2) | 1 (ref) | 1.4 (1.1, 1.7) | 1 (ref) |
|  | Male | 3.8 (2.9, 5.1) | 1.02 (0.81, 1.28) | 2.2 (1.8, 2.7) | 1.14 (0.92, 1.42) | 1.1 (0.7, 1.8) | 0.82 (0.55, 1.22) |
| Schools | Female | 2.9 (2.6, 3.2) | 1 (ref) | 1.8 (1.6, 2.1) | 1 (ref) | 0.9 (0.7, 1.3) | 1 (ref) |
|  | Male | 2.3 (1.8, 2.9) | 0.81 (0.64, 1.00) | 1.5 (1.1, 2.0) | 0.84 (0.66, 1.07) | 0.5 (0.3, 0.7) | **0.48 (0.25, 0.99)** |
| Public spaces | Female | 2.5 (1.8, 3.6) | 1 (ref) | 2.1 (1.5, 2.9) | 1 (ref) | 0.2 (0.1, 0.4) | 1 (ref) |
|  | Male | 1.1 (0.6, 2.0) | **0.45 (0.30, 0.69)** | 0.8 (0.6, 1.3) | **0.42 (0.27, 0.64)** | 0.2 (0.0, 1.0) | 1.24 (0.43, 3.54) |

Adjusted RR: controlled for ethnicity, household socioeconomic deprivation and body weight

Supplementary table 2. Mean rates and rate ratios (95%CI) of snacking, healthful snacking and discretionary snacking by ethnicity and eating location

| Eating location | Gender | All snacks | | Discretionary snacks | | Healthful snacks | |
| --- | --- | --- | --- | --- | --- | --- | --- |
|  |  | Mean rate (95%CI) | Adjusted RR (95%CI) | Mean rate (95%CI) | Adjusted RR (95%CI) | Mean rate (95%CI) | Adjusted RR (95%CI) |
| Homes | NZ European | 4.1 (3.3, 5.0) | 1 (ref) | 2.1 (1.7, 2.6) | 1 (ref) | 1.5 (1.1, 2.0) | 1 (ref) |
|  | Māori | 2.8 (2.0, 3.9) | 0.69 (0.47, 1.01) | 1.7 (1.2, 2.4) | 0.85 (0.53, 1.37) | 0.7 (0.4, 1.4) | **0.47 (0.25, 0.89)** |
|  | Pacific | 3.3 (2.5, 4.4) | 0.89 (0.59, 1.34 | 2.2 (1.7, 2.8) | 1.05 (0.67, 1.65) | 0.7 (0.3, 1.4) | 0.53 (0.27, 1.07) |
| Schools | NZ European | 2.6 (2.1, 3.3) | 1 (ref) | 1.6 (1.2, 2.1) | 1 (ref) | 0.8 (0.5, 1.2) | 1 (ref) |
|  | Māori | 2.6 (2.0, 3.3) | 0.83 (0.62, 1.10) | 2.0 (1.4, 2.8) | 1.14 (0.76, 1.70) | 0.4 (0.3, 0.6) | **0.35 (0.27, 0.47)** |
|  | Pacific | 2.5 (1.9, 3.2) | 0.97 (0.70, 1.36) | 1.5 (1.2, 1.8) | 0.98 (0.68, 1.43) | 0.6 (0.4, 0.9) | 0.62 (0.36, 1.06) |
| Public spaces | NZ European | 1.8 (0.9, 3.3) | 1 (ref) | 1.4 (0.8, 2.4) | 1 (ref) | 0.1 (0.0, 0.9) | 1 (ref) |
|  | Māori | 1.8 (1.2, 2.9) | 0.95 (0.57, 1.59) | 1.5 (1.0, 2.3) | 0.91 (0.59, 1.40) | 0.3 (0.1, 0.8) | **-** |
|  | Pacific | 2.3 (1.4, 3.6) | 1.16 (0.70, 1.92) | 1.9 (1.2, 3.0) | 1.10 (0.67, 1.81) | 0.1 (0.0, 0.4) | 1.78 (0.34, 9.35) |

Adjusted RR: controlled for gender, household socioeconomic deprivation and body weight
